# Combining UAV-based hyperspectral imagery and machine learning algorithms for soil moisture content monitoring

**DOI:** 10.7717/peerj.6926

**Published:** 2019-05-03

**Authors:** Xiangyu Ge, Jingzhe Wang, Jianli Ding, Xiaoyi Cao, Zipeng Zhang, Jie Liu, Xiaohang Li

**Affiliations:** 1 Key Laboratory of Smart City and Environment Modelling of Higher Education Institute, College of Resources and Environment Sciences, Xinjiang University, Urumqi, Xinjiang, China; 2 Key Laboratory of Oasis Ecology, Xinjiang University, Urumqi, Xinjiang, China

**Keywords:** UAV, Precision farming, Hyperspectral imagery, Machine learning

## Abstract

Soil moisture content (SMC) is an important factor that affects agricultural development in arid regions. Compared with the space-borne remote sensing system, the unmanned aerial vehicle (UAV) has been widely used because of its stronger controllability and higher resolution. It also provides a more convenient method for monitoring SMC than normal measurement methods that includes field sampling and oven-drying techniques. However, research based on UAV hyperspectral data has not yet formed a standard procedure in arid regions. Therefore, a universal processing scheme is required. We hypothesized that combining pretreatments of UAV hyperspectral imagery under optimal indices and a set of field observations within a machine learning framework will yield a highly accurate estimate of SMC. Optimal 2D spectral indices act as indispensable variables and allow us to characterize a model’s SMC performance and spatial distribution. For this purpose, we used hyperspectral imagery and a total of 70 topsoil samples (0–10 cm) from the farmland (2.5 × 10^4^ m^2^) of Fukang City, Xinjiang Uygur AutonomousRegion, China. The random forest (RF) method and extreme learning machine (ELM) were used to estimate the SMC using six methods of pretreatments combined with four optimal spectral indices. The validation accuracy of the estimated method clearly increased compared with that of linear models. The combination of pretreatments and indices by our assessment effectively eliminated the interference and the noises. Comparing two machine learning algorithms showed that the RF models were superior to the ELM models, and the best model was PIR (*R*^2^_val_ = 0.907, RMSEP = 1.477, and RPD = 3.396). The SMC map predicted via the best scheme was highly similar to the SMC map measured. We conclude that combining preprocessed spectral indices and machine learning algorithms allows estimation of SMC with high accuracy (*R*^2^_val_ = 0.907) via UAV hyperspectral imagery on a regional scale. Ultimately, our program might improve management and conservation strategies for agroecosystem systems in arid regions.

## Introduction

The soil moisture content (SMC) is a significant physical parameter of soil and a key constraint of soil aggregate structure and nutrient status (Amani et al., 2017; Sadeghi et al., 2017; Wang et al., 2018c). Soil moisture content not only affects the physical and chemical processes of soil but also influences the global ecological environment and hydrological and climate change patterns (Badía et al., 2017; Kumar et al., 2018). Additionally, farmland SMC is an essential parameter for the development of irrigated agriculture. A farmland irrigation system can be more effectively managed when the exact soil moisture status of the farmland is known; moreover, information on farmland SMC can also help improve the soil moisture status at the critical stage of crop growth to improve crop yield and quality (Holzman et al., 2018; Kang et al., 2017; Park et al., 2017). The Xinjiang Uygur Autonomous Region is one of the principal grain producing areas in northwest China. Soil moisture content is the main factor that limits the growth of crops to an oasis in this region. Furthermore, increasing human activities in recent years have led to regional SMC imbalances and increased soil salinization within the oasis (Ma et al., 2018; Wu et al., 2015). During the implementation of sustainable soil management practices and precision agriculture, understanding the spatial distribution of SMC is essential for determining the regional drought situation and measuring water and salt transport in soils. Therefore, obtaining accurate SMC information has important functional significance for the monitoring of crop growth, estimation of production, guidance for rational irrigation decisions, and monitoring of soil drought degree.

The sampling of soils in the field and the oven drying of soils in the lab are well recognized as conventional soil moisture measurement techniques and have been employed as the standard reference for determining SMC (Susha Lekshmi, Singh & Shojaei Baghini, 2014). Nevertheless, these methods can be high cost, low efficiency, and relatively destructive. Compared to common thermogravimetric methods, the rapid development of remote sensing over the last decade, especially of hyperspectral technology, has made it possible to obtain SMC information on a larger scale and with higher efficiency. Researchers have also carried out many constructive explorations (Fabre, Briottet & Lesaignoux, 2015; Hassan-Esfahani et al., 2015; Mouazen & Al-Asadi, 2018; Sadeghi et al., 2017). For example, the spectrum of a vegetation canopy can reflect the growth status and health of vegetation, and its spectral characteristics will change under different soil moisture stress conditions (Holzman, Rivas & Piccolo, 2014). Therefore, unmanned aerial vehicle (UAV)-derived hyperspectral vegetation data could be applied to estimate SMC as an alternative for the accurate assessment of soil moisture.

The spectral index, which is a simple composition of different wavebands, can be used to establish the correlation between spectral data and specific targets to quantitatively estimate hyperspectral information and has become a research hotspot in recent years (Jin et al., 2017b; Marshall & Thenkabail, 2015; Mu et al., 2018). The spectral index of vegetation has two advantages, sensitivity to target parameters and insensitivity to interference factors; thus, the estimation accuracies for specific targets are improved because the effects of interference factors are reduced (Liang et al., 2015). All of the parameters obtained by the canopy spectral index model, including biophysical and biochemical parameters, were found to be strongly correlated with the SMC during an episode of water stress (Wang et al., 2018a). Moreover, different spectral indices are utilized for UAV-based precision farming applications, substantiating the great potential of applying high-resolution UAV data to the agriculture framework to collect and evaluate multispectral images (G, R, near infrared (NIR)) (Jay et al., 2018). However, these types of studies may be more comprehensive if the pretreatment of data is considered. These spectral indices are based mainly on the original spectral reflectance. Unpretreated data are a combination of several composite signals with various overlapping data. This type of data reflects only specific spectral information and is difficult to data-mine effectively and efficiently. To rectify this problem, pretreated data are introduced to eliminate external noise, enhance spectral features, boost nonlinear relations, and improve the accuracy of specific target estimation models (Ding et al., 2018; Gobrecht et al., 2016; Nawar et al., 2016). Furthermore, simple spectral indices consider only the interaction between the spectrum and object, without regarding the interaction between the reflectance spectrum. Hence, the optimization of spectral indices using the 2D correlation coefficient could detect more feature wavelengths and further enhance the correlations between specific properties and spectral characteristics of a target.

Mathematical models are a common strategy used to estimate SMC via hyperspectral reflectance data, particularly linear regression models that include partial least squares regression (PLSR) (Nawar et al., 2014; Xu et al., 2016; Yu et al., 2016). However, linear regression models also need improvement because the relationship between spectral parameters and soil attributes is rarely linear in nature. Machine learning algorithms are alternative approaches to this problem (Nawar & Mouazen, 2017). The neural network algorithm is a widely implemented machine learning algorithm. The precision of the extreme learning machine (ELM) developed by Huang (Huang, Zhu & Siew, 2006) was estimated in forecasting *SM*-derived data. The ELM is a relatively novel algorithm among neural network algorithms. Compared to other neural network algorithms, ELM is a simple and fast algorithm with outstanding generalization and migration (Khosravi et al., 2018). Extreme learning machine has gradually gained popularity in quantitative remote sensing studies, especially in solving regression and classification problems (Huang et al., 2015; Maimaitijiang et al., 2017; Morellos et al., 2016). Meanwhile, numerous studies have reported that the random forest (RF) method is more likely to provide spectral estimations than are methods via PLSR (Douglas et al., 2018; Wang et al., 2018b). The RF method is an outstanding ensemble-learning algorithm. It has been proven to be superior to Cubist, artificial neural networks, and support vector machines in modeling performance (Gomes et al., 2019; Peng et al., 2019; Nawar & Mouazen, 2017; Zeraatpisheh et al., 2019). Its advantages are overcoming redundant information while implemented on high-dimensional data (Belgiu & Drăguţ, 2016) and presenting generally improved precision, accuracy, and efficiency (Ding et al., 2018). Furthermore, the RF algorithm is a robust method for building an estimation model with a small sample size (Lindner et al., 2015). It is obvious that the RF approach can better process many input variables as well as nonsymmetrical datasets.

Technical advancements in the field of remote sensing have ignited prosperity in the UAV field which provide images with high spatial resolution. Moreover, the flexibility of UAV allows them to contribute to data collection in a variety of fields rather than being constrained to fields with specific soil conditions (Jin et al., 2017a). Unmanned aerial vehicles are generally utilized as a remote sensing platform in a series of environmental resource applications. The images collected from various sensors have been widely applied to collect agricultural information (Adão et al., 2017; Gevaert et al., 2015), such as biophysical and biochemical vegetation parameters (Schirrmann et al., 2016) and soil physical and chemical properties (Guo et al., 2019). Although several studies have predicted the attributes of vegetation or soil based on UAV images, estimations of SMC via vegetation canopy data are not often reported.

The major objectives of this study are to (1) explore the relationship between the SMC and various hyperspectral 2D indices based on different pretreatment methods, (2) develop a hyperspectral quantitative estimation model of SMC in oasis farmland in arid area through two machine learning algorithms based on 2D spectral indices, and (3) attempt to digitally map UAV hyperspectral imagery to predict SMC in topsoil of arid agriculture areas.

## Materials and Methods

### Study area

**Figure 1:**
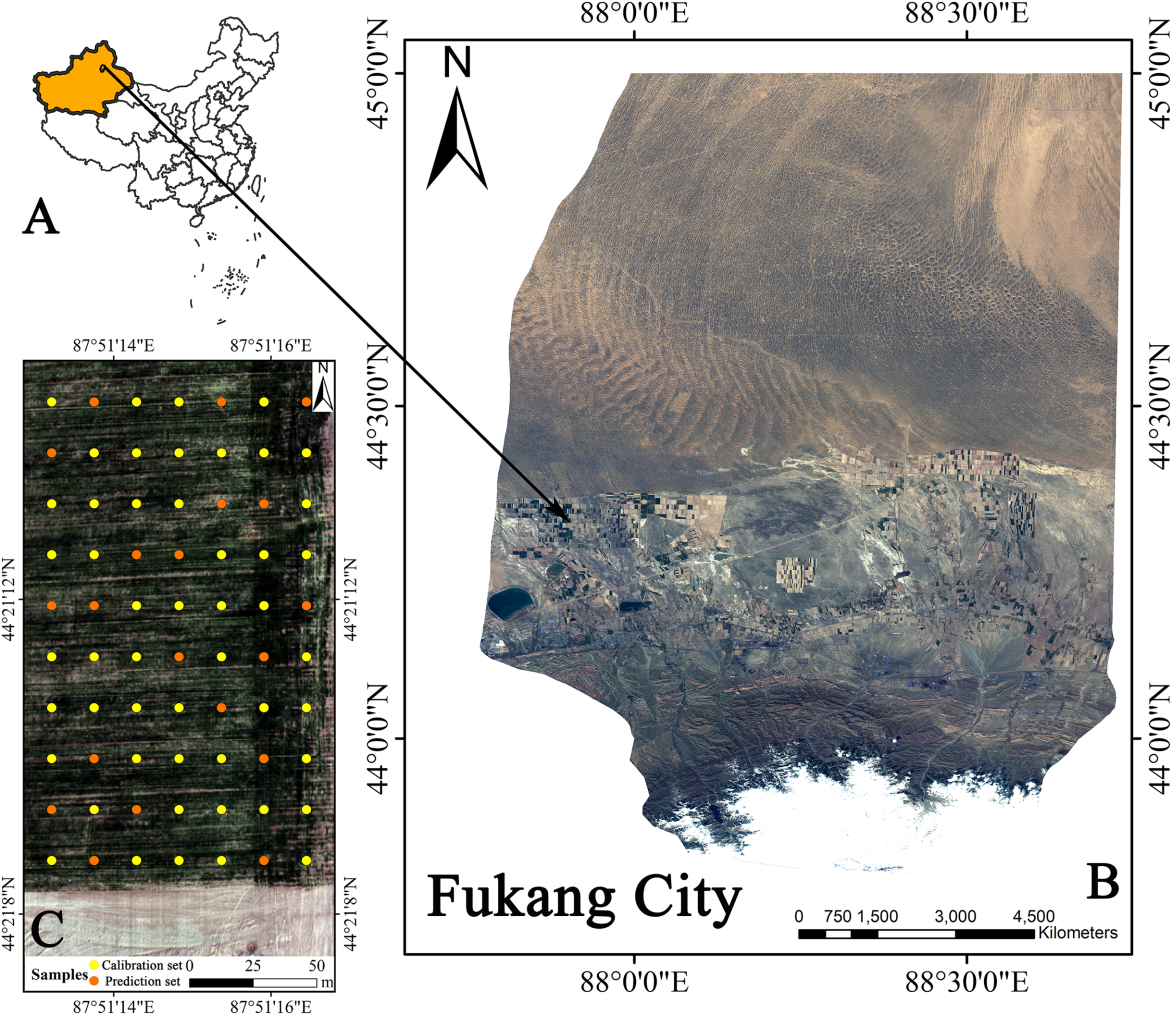
Geographical location of Fukang City and the distribution of sampling sites. (A) Xinjiang’s position in China. (B) Fukang City. (C) Sampling point schematic (Map credit: Xiangyu Ge).

The field selected in this study was in Fukang City, Xinjiang Uygur Autonomous Region, China (87°51′15″E, 44°21′14″N). This area is located in the transition zone of the Gurban Tongut Desert along the northern margin of the Fukang Oasis (Fig. 1). The study area has a typical temperate continental desert climate with an average annual precipitation of less than 200 mm with uneven distribution. The annual average temperature is approximately 7.1 °C. The annual frost-free period can reach 175 days, and the harvest principle is one harvest per year. The crops grown in the field are winter wheat.

### UAV remote sensing data acquisition

**Figure 2:**
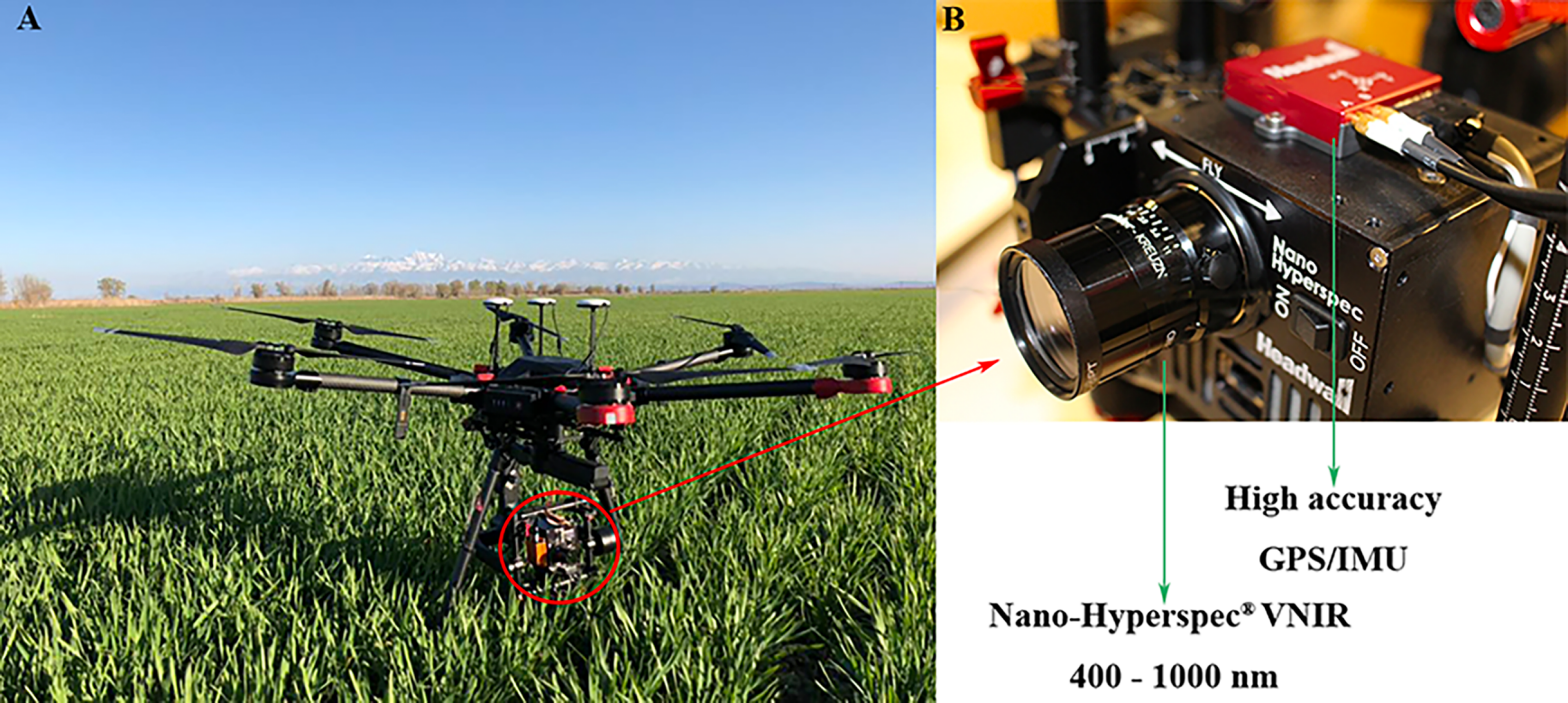
UAV platform and airborne imaging hyperspectral sensor. (A) UAV. (B) Hyperspectral sensor (Photograph credit: Xiangyu Ge).

The flight platform selected in this study was the DJI Matrice 600 Pro^®^ (Shenzhen Dajiang Innovation Technology Co., Ltd., China), which is a six-rotor UAV equipped with the Headwall Nano-Hyperspec^®^ hyperspectral sensor (Headwall Photonics Inc., Bolton, MA, USA) (Fig. 2). The Nano-Hyperspec airborne hyperspectral imaging spectrometer has a band range of 400–1,000 nm, a spectral resolution of six nm, a resampling interval of 2.2 nm, 270 spectral bands and 640 spatial bands in the visible and near infrared (VIS-NIR). The feature of full-frame imaging in the interval, combined with the GPS and inertial measurement unit module, can simultaneously acquire the real-time altitude information of the UAV. At a height of 100 m, the Nano-Hyperspec sensor with a focal length of 12 mm captures 640 × 480 pixels of hyperspectral imagery with a spatial resolution of approximately four cm. In this study, there was no precipitation or artificial interference within 5 days before field work to ensure the objectivity of the data. UAV remote sensing data were acquired on April 17, 2018 (the reviving period of winter wheat). Hyperspectral images were collected over the field at 15:00 Beijing time. The weather was clear and windless, and the field of vision was good. Dark current correction and whiteboard calibration were performed on the sensor before take-off. After data acquisition, data postprocessing and orthorectification were performed using Hyperspec III and Headwall SpectralView software.

### SMC data acquisition

**Figure 3:**
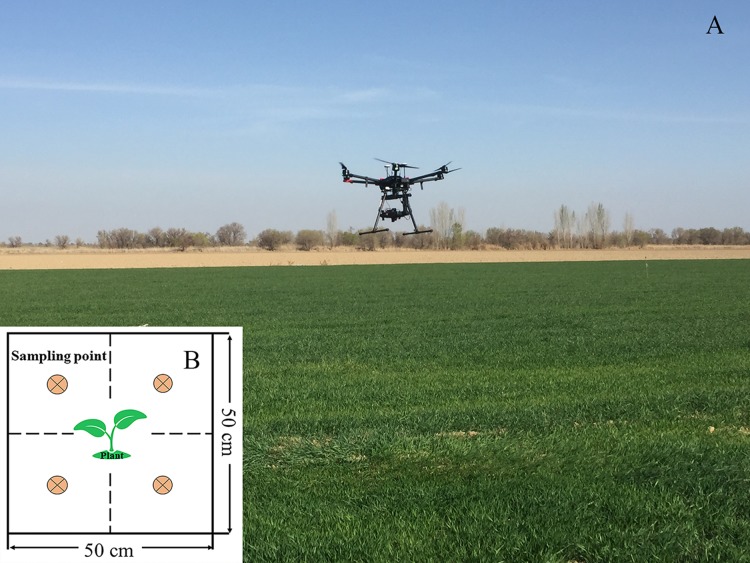
Application scene of UAV over the cropland and sampling cells. (A) Application scene of UAV. (B) Four-point method of sampling (Photograph credit: Xiangyu Ge).

The soil samples were collected simultaneously with the UAV air operations, and 70 sampling cells (0.5 m × 0.5 m) (Fig. 3) were uniformly collected from the farmland; the position of each sampling area was recorded by GPS. The soil samples of each point were collected by using the four-point method around wheat plants. The sampling depth was 0–10 cm, and the soil samples were sealed and stored in an aluminum box. During laboratory processing, the samples from the aluminum box were oven-dried indoors (105 °C incubator, 48 h) to obtain 70 SMC data samples to construct the SMC hyperspectral quantitative estimation model and verify its accuracy.

### Data processing

Hyperspectral data preprocessing is essential for deep mining of spectral data and thus improved modeling accuracy (Li et al., 2015). A spectrometer consists mainly of photoelectric conversion, transmission, and processing systems. Each module inside generates noise to varying degrees, and the real spectral information of the ground object is inevitably affected by noise, which needs to be detected and removed (Jin et al., 2016). Therefore, this study smoothed the hyperspectral images based on the Savitzky–Golay (SG) filter (second order polynomial smoothing and 5-band window widths). The SG in this study was performed in MATLAB software version R2016b (MathWorks, Natick, MA, USA).

First-derivative (FD), second-derivative (SD), absorbance (A), continuum-removal (CR) are effective preprocessing methods that are important spectral significance in the field of spectral analysis because they can eliminate background noise to some extent (Cheng et al., 2019). These methods enhance spectral absorption and reflection characteristics (Liu & Han, 2017; Žížala, Zádorová & Kapička, 2017). Effective pretreatment helps capture subtle differences in spectral data and improves the estimation accuracy of surface parameters. In this paper, the SG filtered image was used as the pretreated base image (R), and six preprocessing methods were performed: first-derivative R (FDR), second-derivative R (SDR), CR, A, first-derivative absorbance (FDA), and second-derivative absorbance (SDA). These methods were conducted based on the ENVI/IDL 5.3 platform (Harris Geospatial, Melbourne, FL, USA). The average of the spectral data in each sampling cell were extracted to prepare for the construction of spectral indices and modeling.

### Spectral indices construction

#### Common spectral indices

The spectral index method has advantages of both eliminating the environmental background noise and having more obvious sensitivity than a single band. To ensure an optimal band combination in the hyperspectral data when utilizing the vegetation canopy spectral information, this study selected 30 widely applied spectral indices to represent the SMC, as shown in Table 1. Difference indices, ratio indices, normalized indices, and perpendicular indices, as well as some modified indices, enhanced indices, and red edge indices, were included among the selected indices.

**Table 1:** Common spectral indices.

Indices	Formulations	References
NDVI	(*R*_800_ − *R*_680_)/(*R*_800_ + *R*_680_)	(Haboudane et al., 2004)
NDVI705	(*R*_750_ − *R*_705_)/(*R*_750_ + *R*_705_)	(Sims & Gamon, 2002)
RVI	*R*_800_/*R*_680_	(Sims & Gamon, 2002)
NDCI	(*R*_762_ − *R*_527_)/(*R*_762_ + *R*_527_)	(Liang et al., 2015)
GNDVI	(*R*_750_ − *R*_550_)/(*R*_750_ + *R*_550_)	(Yao et al., 2017)
OSAVI	[(1 + 0.16)(*R*_800_ − *R*_670_)]/(*R*_800_ + *R*_670_ + 0.16)	(Haboudane et al., 2002)
NDRE	(*R*_740_ − *R*_705_)/(*R*_740_ + *R*_705_)	(Broge & Leblanc, 2001)
mNDVI705	(*R*_750_ − *R*_705_)/(*R*_750_ + *R*_705_ + 2*R*_445_)	(Liang et al., 2015)
VOG1	*R*_740_/*R*_720_	(Vogelmann, Rock & Moss, 1993)
VOG3	(*R*_734_ − *R*_747_)/(*R*_715_ + *R*_720_)	(Vogelmann, Rock & Moss, 1993)
VOG2	(*R*_734_ − *R*_747_)/(*R*_715_ + *R*_726_)	(Vogelmann, Rock & Moss, 1993)
CARI	(*R*_700_ − *R*_670_)/0.2(*R*_700_ + *R*_670_)	(Main et al., 2011)
MTVI1	1.2[1.2(*R*_800_ − *R*_550_) − 2.5(*R*_670_ − *R*_550_)]	(Haboudane et al., 2004)
TVI	0.5[120(*R*_750_ − *R*_550_) − 2.5(*R*_670_ − *R*_550_)]	(Broge & Leblanc, 2001)
DVI	*R*_800_ − *R*_680_	(Tian et al., 2011)
RDVI	(*R*_800_ − *R*_670_)/(*R*_800_ + *R*_670_)^0.5^	(Yao et al., 2017)
SPVI	1.48(*R*_800_ − *R*_670_) − 1.2|*R*_530_ − *R*_670_|	(Main et al., 2011)
WI/NDVI	(*R*_900_/*R*_970_)/[(*R*_800_ − *R*_680_)/(*R*_800_ + *R*_680_)]	(McCall et al., 2017)
EVI	2.5(*R*_800_ − *R*_670_)/(*R*_800_ − 6*R*_670_ − 7.5*R*_475_ + 1)	(Huete et al., 1997)
NVI	(*R*_777_ − *R*_747_)/*R*_673_	(Gupta, Vijayan & Prasad, 2001)
MSAVI	0.5(2*R*_800_ + 1 − [(2*R*_800_ + 1)^2^ − 8(*R*_800_ − *R*_670_)]^0.5^)	(Tian et al., 2011)
WI	*R*_900_/*R*_970_	(Peñuelas et al., 1993)
REP	700 + [40(*R*_670_ + *R*_780_)/2 − *R*_700_]/(*R*_740_ − *R*_700_)	(Gupta, Vijayan & Prasad, 2001)
PRI	(*R*_531_ − *R*_570_)/(*R*_531_ + *R*_570_)	(Sims & Gamon, 2002)
MTVI2	$\displaystyle{{1.5\left[ {1.2\left( {{R_{800}} - {R_{550}}} \right) - 2.5\left( {{R_{670}} - {R_{550}}} \right)} \right]} \over {{{\left[ {{{\left( {2{R_{800}} + 1} \right)}^2} - \left( {6{R_{800}} - 5\sqrt {{R_{670}}} } \right) - 0.5} \right]}^{0.5}}}}$	(Yao et al., 2017)
TCARI2	3[*R*_750_ − *R*_705_ − 0.2(*R*_750_ − *R*_550_)(*R*_750_/*R*_705_)]	(Wu et al., 2008)
TCARI/OSAVI	TCARI/OSAVI	(Wu et al., 2008)
MCARI/OSAVI	MCARI/OSAVI	(McCall et al., 2017)
TCAR1	3[(*R*_700_ − *R*_670_) − 0.2(*R*_700_ − *R*_550_)(*R*_700_/*R*_670_)]	(Haboudane et al., 2002)
MCARI	[(*R*_700_ − *R*_670_) − 0.2(*R*_700_ − *R*_550_)(*R*_700_/*R*_670_)]	(Haboudane et al., 2002)

#### Construction of 2D spectral indices

To fully exploit the spectral data, this study selected the difference index (DI), the ratio index (RI), the normalized difference index (NDI) (Hong et al., 2018; Wang et al., 2018d), and the perpendicular index (PI) based on previous studies. Four spectral indices were used to estimate the optimal band for SMC. The mathematical expression of these indices were as follows:

(1) $${\rm{D}}{{\rm{I}}_{\left( {{R_i},\;{R_j}} \right)}} = {R_i} - {R_j}$$

(2) $${\rm{R}}{{\rm{I}}_{\left( {{R_i},\;{R_j}} \right)}} = {R_i}/{R_j}$$

(3) $${\rm{ND}}{{\rm{I}}_{\left( {{R_i},\;{R_j}} \right)}} = \left( {{R_i} - {R_j}} \right)/\left( {{R_i} + {R_j}} \right)$$

(4) $${\rm{P}}{{\rm{I}}_{\left( {{R_i},\;{R_j}} \right)}} = \left( {{R_i} - 0.4401{R_j} - 0.3308} \right)/\left( {\sqrt {1 + {{0.4401}^2}} } \right)$$

where *R*_*i*_ and *R*_*j*_ are the spectral reflectance of *i* and *j*, which were arbitrarily acquired within the operating range of the hyperspectral sensor (400–1,000 nm). The constant term in the PI calculate was based on the soil line coefficient of the UAV imagery (In this study, the two-dimensional spectral space of red-NIR from pure soil pixels was selected to extract the soil line in which the red band was *R*_655_, NIR band was *R*_866_. The soil line was: $y = 0.4401x + 0.3308$). The correlation between the two and the optimal index was determined using MATLAB R2016b.

### Model calibration, evaluation, and comparison

In this study, sample partitioning was based on the joint x–y distance (SPXY) algorithm (Ulissi et al., 2011). 50 samples were selected as the calibration set and 20 samples were used as the prediction set. The SPXY algorithm was conducted via MATLAB R2016b. To compare the common spectral indices, the linear fit between several spectral indices and SMC was calculated. The calibration set was used as the source for the fitting equation and the validation set is used to assess the precision of the fitting result. The estimated SMC was modeled based on the RF and ELM algorithms, and seven optimal spectral indices and measured SMC values were used as the independent and response variables, respectively.

#### Extreme learning machine

Extreme learning machine is a new effective neural network algorithm that was developed from the feed-forward neural network (Guang-Bin, Qin-Yu & Chee-Kheong, 2004). Technically, ELM is an ordinal neural network algorithm with single-hidden-layer feed-forward features and was designed by Huang for regression and classification (Huang et al., 2012). Unlike a general neural network, ELM avoids the need to manually set many parameters. The only required parameter is the number of hidden nodes (Huang, Zhu & Siew, 2006). With its rapid learning ability, outstanding generalization, and convenient parameter setting, ELM overcomes the defects of traditional neural networks, including inappropriate learning rates and local optimal solutions. During the training process, the input weights of the iterative network and the offset of the hidden elements are avoided, and the optimal solution can be obtained. In this study, the ELM algorithm was conducted via MATLAB R2016b. The hidden layer nodes were set to 30, and the sigmoid function was selected as the activation function.

#### Random forest

Random forest regression is a popular machine learning algorithm that possesses ideal estimation capability, especially for high-dimensional datasets (Belgiu & Drăguţ, 2016; Mutanga, Adam & Cho, 2012). Random forest regression is also an ensemble-learning algorithm based on a classification and regression tree (Ließ, Glaser & Huwe, 2012). Random forest regression is good at fitting data through a set of decision tree models (Hong et al., 2019). The trees are built using a subset of samples from the training samples that are replaced. The design of such an algorithm makes full use of the samples, and some samples will even be selected multiple times, so it unlikely that data will remain. For each tree node and split point, the data are recursively divided into nodes, and the split points are based on the values of the predictors, which improve the predictability of the response variables. The major parameters in this study were set as follows: the number of trees was 500, the minimum number of nodes (*nodesize*) was 5, and the number of features tried at each node (*mtry*) depended on the lowest out-of-bag error. The RF algorithm was conducted via MATLAB R2016b.

#### Model evaluation and comparison

To quantify the performance of spectroscopic models based on RF and ELM, the effect of the models was assessed utilizing the determination coefficients (*R*^2^), the root mean squared error (RMSE), and the relative percent deviation (RPD). The formulas and definitions were given by Nocita et al. (2013). In our research, *R*^2^ included estimated values against the SMC values in the calibration set (*R*^2^_cal_) and estimated values against the SMC values in the validation set (*R*^2^_val_). Root mean squared error included the RMSE of calibration (RMSEC) and the RMSE of validation (RMSEP). According to Qi et al. (2018), it is feasible to adopt three categories of criteria to assess model predictability: category I (RPD > 2.0) with excellent predictability; category II (1.4 < RPD < 2.0) with moderate predictability; and category III (RPD < 1.4) with poor predictability.

**Figure 4:**
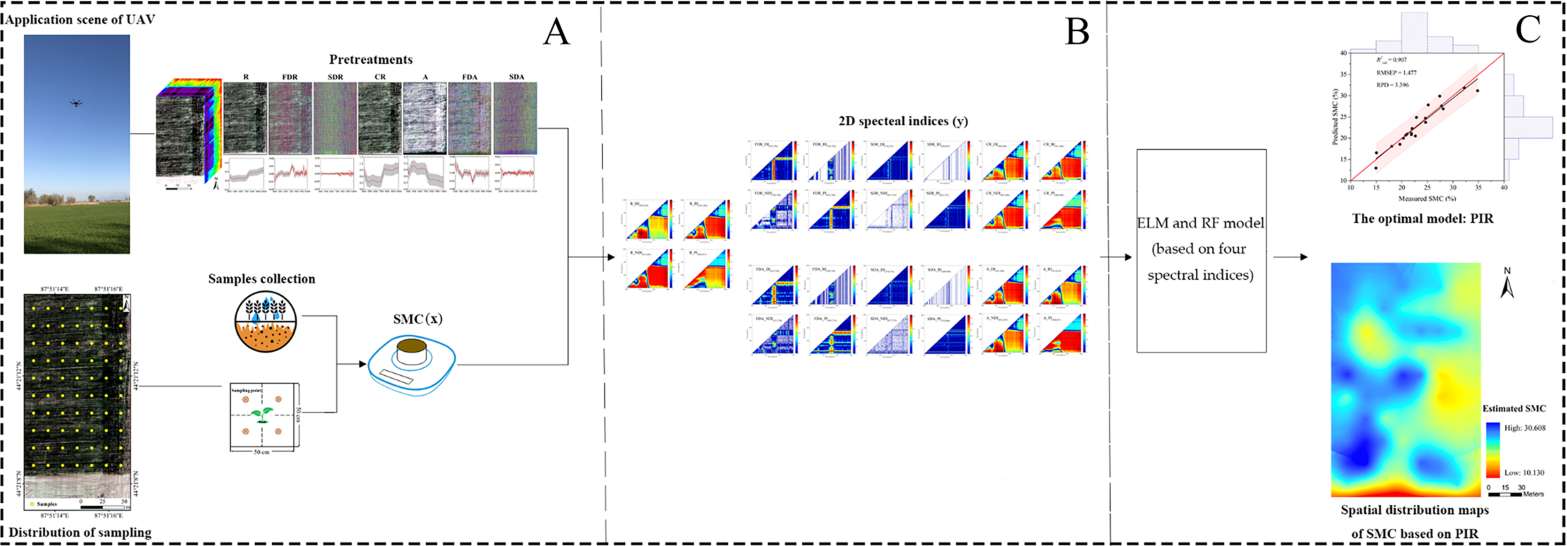
Flowchart of the study procedure. Flowchart of the study procedure: (A) Data collection and pretreatments (Photograph credit: Xiangyu Ge); (B) Construction of 2D spectral indices based on DI, RI, NDI and PI; (C) Comparison of model and determination of SMC based on the optimal model and spatial distribution map using PIR.

The steps of SMC estimation are illustrated in Fig. 4.

## Result

### Descriptive statistical analysis

**Figure 5:**
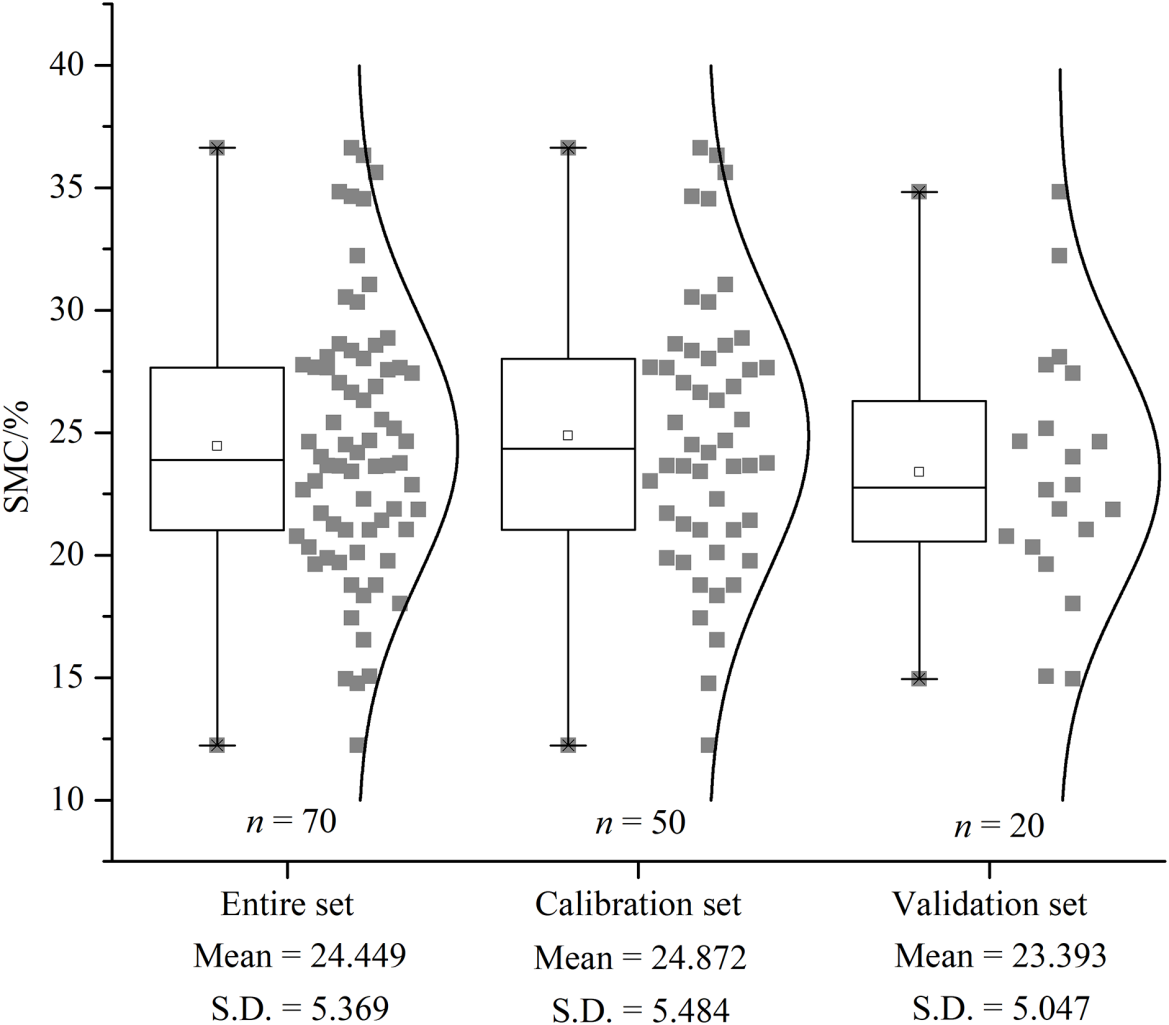
The descriptive statistical results of SMC. Box plot and distribution of SMC for the whole, calibration, and validation datasets. S.D. indicates standard deviation.

Descriptive statistical results were presented for the entire dataset as well as the calibration and validation sets (Fig. 5). The average SMC in the entire set was 24.45%, with a standard deviation (SD) of 5.37%. The surface soil moisture was affected by the environment in the area where the same crop was planted. The average SMCs of the calibration (12.23–36.63%) and validation (14.95–34.83%) sets were 24.87% and 23.39%, respectively. The similar SD and mean values indicated that the distribution of the SMC of all datasets was the standardized normal distribution with similar statistical characteristics. The calibration and validation sets via the SPXY algorithm maintained a statistical distribution analogous to the entire set of SMC. To ensure representative samples, potentially biased estimates in the calibration and validation set were excluded.

**Figure 6:**
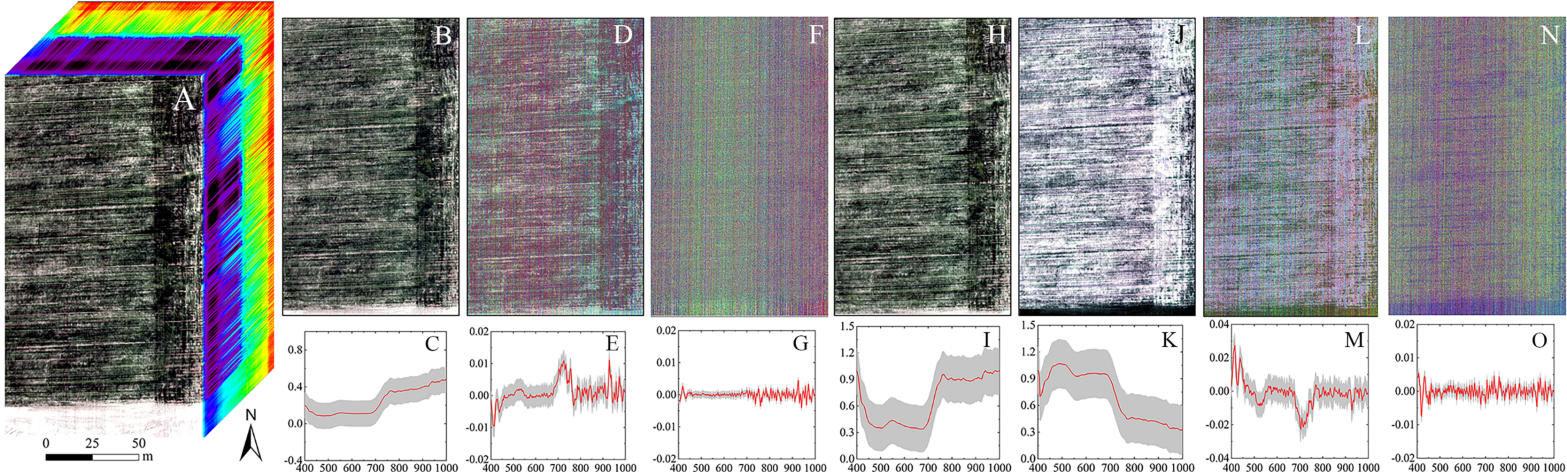
The hyperspectral imageries and spectral curves based on different pretreatments. (A) Hyperspectral image cube. (B) Image based on R. (C) Spectral curve based on R. (D) Image based on FDR. (E) Spectral curve based on FDR. (F) Image based on SDR. (G) Spectral curve based on SDR. (H) Image based on CR. (I) Spectral curve based on CR. (J) Image based on A. (K) Spectral curve based on A. (L) Image based on FDA. (M) Spectral curve based on FDA. (N) Image based on SDA. (O) Spectral curve based on SDA. The hyperspectral images and spectral curves based on different pretreatments (the red line represents the average spectrum and the gray region represents the standard deviation). The images are RGB images, where the red, green and blue bands are *R*_659_, *R*_550_, and *R*_479_, respectively.

In this study, pretreatments had different effects on the hyperspectral imageries (Fig. 6). As the order of the derivative increased, the intensity of the processed spectrum decreased, considering the *y*-axis scales from FDR to SDR. A and CR enhanced the spectral intensity of some bands and especially highlighted blue band and red edge information.

### Appropriate spectral indices for SMC estimation

In the SMC estimation model based on these 30 spectral indices, the model calibration set yielded higher RMSE and lower *R*^2^ values than did the validation set (Table 2). This result indicated that the estimated model fit was poor and that the independent variables in the model were inadequate for explaining the dependent variable. In addition, collinearity of the independent variables would also yield this result. To more intuitively display the SMC estimation accuracy of the indices, the indices were sorted in accordance with *R*^2^ in descending order. The order of the sorting was basically the same as when sorted by the correlation coefficients (*r*) of the spectral index and the SMC. NDVI (*R*^2^ = 0.664), NDVI_705_ (*R*^2^ = 0.663), and RVI (*R*^2^ = 0.662) presented the three highest rankings, demonstrating that these three spectral indices were highly correlated with SMC. The normalized indices were ranked in the top row, followed by the RI. However, the predictability of the model indicated that the index models with higher *R*^2^ values had lower RPD values. NDVI possessed poor predictability (RPD = 0.871), but similar to MCARI (*R*^2^ = 0.153, RPD = 5.366) and TCAR1 (*R*^2^ = 0.153, RPD = 5.366), this index model yielded lower *R*^2^ with higher RPD values. Technically, the *R*^2^ values indicated that all models had difficulty meeting the needs of model estimation. Therefore, the estimation of SMC by hyperspectral indices was ambiguous in this study area.

**Table 2:** The fitting equations and their accuracies estimated by common spectral indices.

Indices	*r*	Fitting equation	*R*^2^_cal_	RMSEC	*R*^2^_val_	RMSEP	RPD
NDVI	0.466	*y* = −30.66x+41.791	0.398	4.203	0.664	3.154	0.871
NDVI705	0.465	*y* = −33.709x+36.614	0.398	4.203	0.663	3.170	0.901
RVI	0.461	*y* = −2.984x+36.058	0.399	4.200	0.662	3.247	0.987
NDCI	0.466	*y* = −44.116x+49.425	0.401	4.195	0.659	3.181	0.847
GNDVI	0.452	*y* = −37.084x+42.524	0.382	4.258	0.654	3.196	0.897
OSAVI	0.437	*y* = −31.458x+40.177	0.368	4.308	0.652	3.280	0.986
NDRE	0.457	*y* = −38.105x+36.717	0.393	4.222	0.649	3.233	0.918
mNDVI705	0.477	*y* = −27.917x+41.049	0.418	4.132	0.643	3.188	0.830
VOG1	0.431	*y* = −19.775x+53.052	0.365	4.318	0.633	3.326	0.988
VOG3	0.422	*y* = 79.265x+32.097	0.351	4.366	0.625	3.279	0.933
VOG2	0.424	*y* = 69.876x+31.809	0.353	4.358	0.622	3.288	0.938
CARI	0.378	*y* = −21.331x+39.558	0.308	4.506	0.605	3.523	1.180
MTVI1	0.354	*y* = −24.908x+35.067	0.292	4.560	0.604	3.709	1.469
TVI	0.325	*y* = −0.799x+35.303	0.263	4.652	0.584	3.826	1.649
DVI	0.348	*y* = −40.556x+35.793	0.289	4.567	0.583	3.769	1.515
RDVI	0.355	*y* = −41.61x+46.336	0.293	4.556	0.580	3.705	1.388
SPVI	0.348	*y* = −27.198x+35.464	0.289	4.568	0.575	3.761	1.466
WI/NDVI	0.414	*y* = 10.046x+7.791	0.371	4.297	0.540	3.623	0.870
EVI	0.062	*y* = −18.21x+31.547	0.372	4.292	0.524	3.759	1.206
NVI	0.406	*y* = −18.21x+31.547	0.372	4.292	0.524	3.759	1.206
MSAVI	0.273	*y* = −16.695x+21.407	0.227	4.764	0.509	4.156	2.170
WI	0.238	*y* = −45.663x+65.717	0.177	4.915	0.474	4.131	2.022
REP	0.343	*y* = 0.162x+−98.259	0.305	4.516	0.460	3.936	0.987
PRI	0.364	*y* = −198.981x+16.402	0.332	4.427	0.449	3.898	1.157
MTVI2	0.018	*y* = −0.041x+24.633	0.021	5.360	0.432	5.222	0.889
TCAR2	0.253	*y* = −47.674x+35.662	0.201	4.842	0.430	4.141	1.782
TCARI/OSAVI	0.350	*y* = 113.241x+5.231	0.336	4.414	0.419	4.204	0.910
MCARI/OSAVI	0.350	*y* = 339.723x+5.231	0.336	4.414	0.419	4.204	0.910
TCAR1	0.068	*y* = −88.168x+31.782	0.052	5.275	0.153	4.969	1.366
MCARI	0.068	*y* = −264.505x+31.782	0.052	5.275	0.153	4.969	1.366

**Figure 7:**
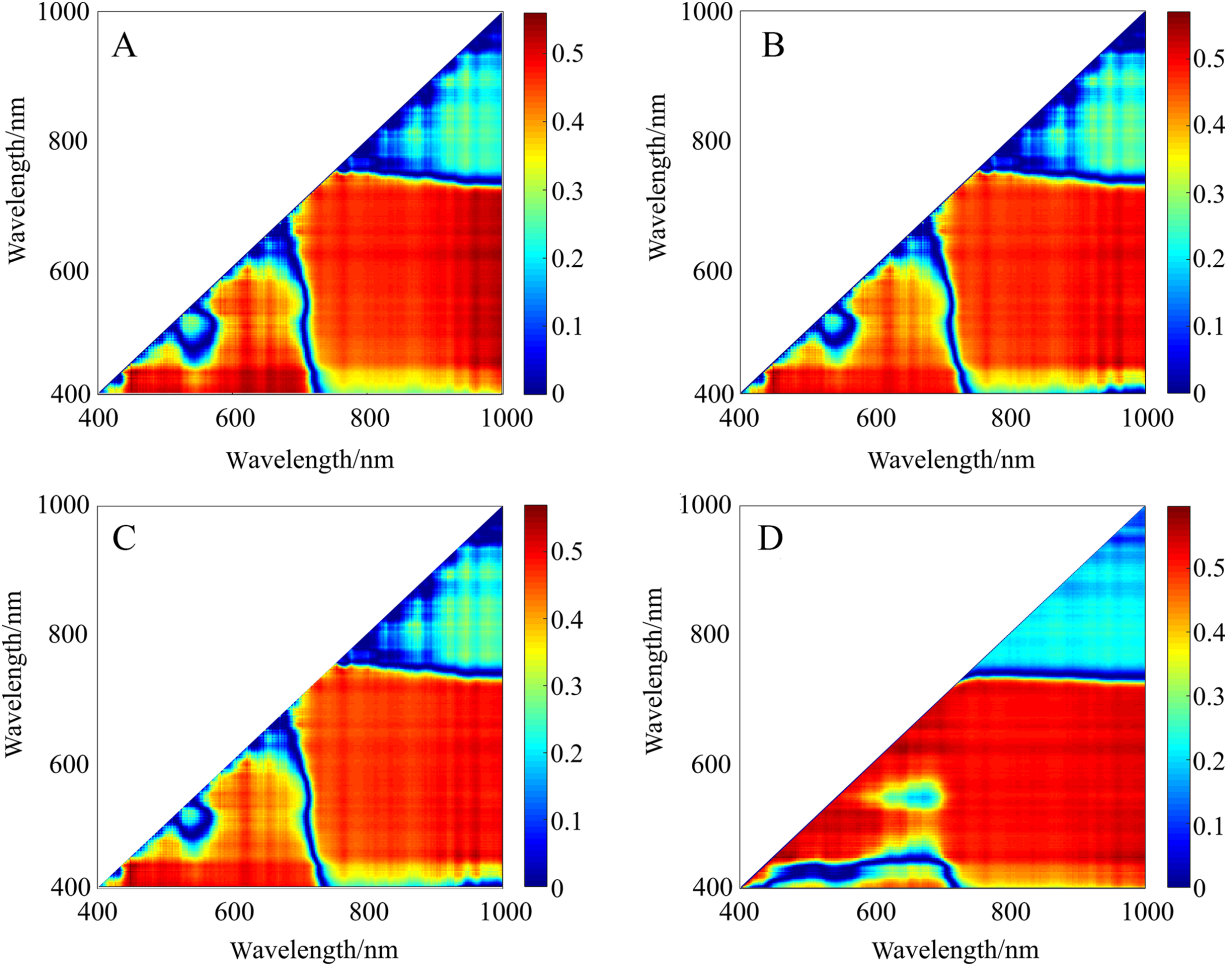
*r*^2^ maps of 2D optimal spectral indices based on different pretreatments. (A) *r*^2^ maps of A_DI_(431,446)_. (B) *r*^2^ maps of CR_NDI_(431,446)_. (C) *r*^2^ maps of CR_RI_(431,446)_. (D) *r*^2^ maps of A_PI_(446,471)_. The colorbar illustrates the value of the square of the correlation coefficient (*r*^2^) between SMC and spectral indices, and the *x*-axes and *y*-axes indicate the wavebands of 400–1,000 nm. Dark red portrays a high *r*^2^ between SMC and the spectral indices. To improve the comparison, *r*^2^ was converted into the absolute value of the correlation coefficient (|*r*|) to evaluate its validity.

The correlativity between SMC and 2D spectral indices (DIs, RIs, NDIs, and PIs) for varying spectral transformations in the calibration set was further explored (Fig. 7), and detailed results are provided in Figs. S1–S7. The results substantiate that the 28 spectral indices established in this study with SMC all passed the significance test at the 0.01 level (threshold value was ±0.306) (Table 3). For the unpretreated spectral data, which had strong sensitivity compared to traditional indices, the |*r*| distribution of the constructed DI, RI, NDI, and PI ranged from 0.724 to 0.772 (greater than 0.664). Nonetheless, the SMC was more sensitive to spectral indices of different pretreatments than to unpretreated spectral data. Thereinto, the |*r*| of the A-DI, A-PI, CR-NDI, and CR-RI was above 0.748, which was optimal. Different pretreatment schemes improved the correlation between spectral indices and SMC to varying degrees, and the optimal index was A-PI (*r* = 0.788).

**Table 3:** |*r*| between SMC and spectral indices based on different pretreatments.

Spectral indices	Pretreatment method						
	R	FDR	SDR	CR	A	FDA	SDA
DI	0.724	0.662	0.551	0.737	0.748	0.742	0.577
NDI	0.748	0.674	0.487	0.755	0.725	0.616	0.561
RI	0.747	0.668	0.475	0.755	0.720	0.624	0.424
PI	0.772	0.693	0.554	0.746	0.773	0.738	0.569

### Construction of estimation models

The indices (DIs, RIs, NDIs, and PIs) used for modeling in the paper were the most relevant in different pretreatments. The models constructed by the two algorithms were compared (Table 4), which indicated that the prediction model based on RF performed better and possessed superior *R*^2^_val_ (0.847–0.907) and RPD (2.867–3.396) and inferior RMSEP (1.477–1.665) values did than the model based on ELM, no matter which spectral indices were used. For the RF model, the PI had the highest *R*^2^_val_ (0.907) and RPD (3.396) and the lowest RMSEP (1.477). The worst RF predicting model had an *R*^2^_val_ of 0.847, but the best ELM model had an *R*^2^_val_ of only 0.820. Additionally, the values from the ELM calibration set were higher than those from the validation set, ranging between 0.781 and 0.823. This result indicated that the modeling effect was improper.

**Table 4:** Calibration and validation results for SMC estimation based on different modeling strategies.

Model	*R*^2^_cal_	RMSEC	*R*^2^_val_	RMSEP	RPD	Abbreviations
PI_RF	0.896	1.768	0.907	1.477	3.396	PIR
NDI_RF	0.856	2.104	0.872	1.479	3.245	NDIR
DI_RF	0.832	2.310	0.852	1.665	2.908	DIR
RI_RF	0.828	2.367	0.847	1.606	2.867	RIR
RI_ELM	0.823	2.301	0.820	1.984	2.322	RIE
PI_ELM	0.823	2.351	0.817	2.196	2.435	PIE
NDI_ELM	0.824	2.302	0.815	2.277	2.389	NDIE
DI_ELM	0.781	2.566	0.774	2.087	2.220	DIE

**Figure 8:**
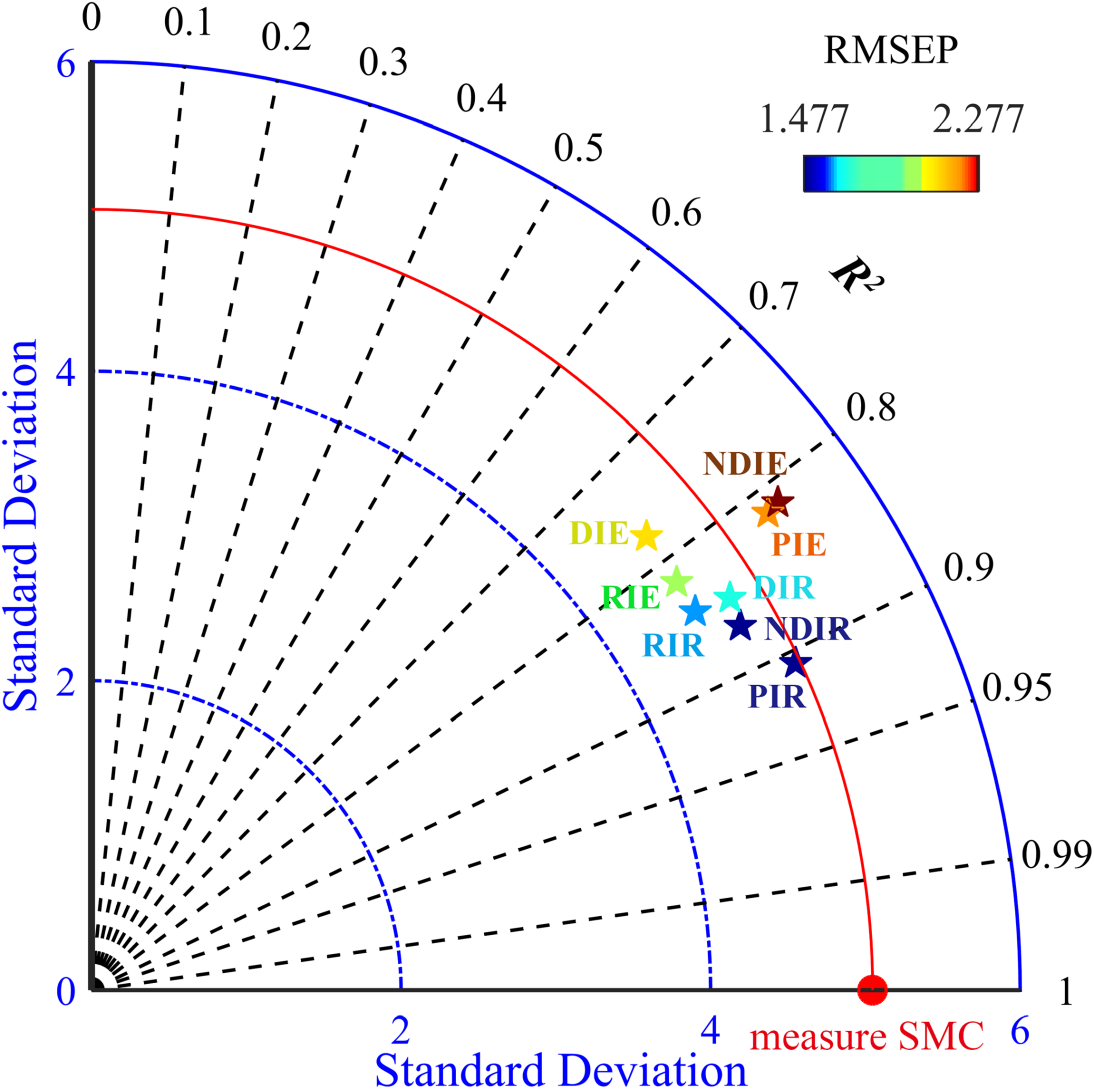
Taylor diagram showing the performance of the evaluated models. The black line indicates *R*^2^_val_, the blue line indicates the SD, and the colorful pentagrams represent the eight models, whose colors from dark blue to deep red indicate small to large RMSEP values. The red line represents the measured SMC.

To better explain the model prediction effect, this study introduced a Taylor diagram (Guevara et al., 2018). The closer the pentagram was to this line, the closer the model prediction was to the measured SMC and the more similar statistical characteristics that is possessed (Fig. 8). Overall, the RF model was closer to the red line than the ELM model, while the PIR was the closest and the DIE was the farthest. A comparison of the closeness illustrated that the ranking of the predictive performance was PIR > DIR > NDIR > PIE > NDIE > DIE > RIE > RIR. The RMSE values of the RF model were all smaller than those of the ELM model. NDIE was dark red to indicate that its RMSE value was the largest, and PIR was dark blue to indicate that its value was the smallest. Moreover, all the RF models were closer to the horizontal black line indicating that they possess *R*^2^ close to 1. Therefore, the models constructed with PI performed the best, and the models constructed with RI performed the worst. The best two-dimensional spectral index model in this study was PIR.

### Digital mapping

**Figure 9:**
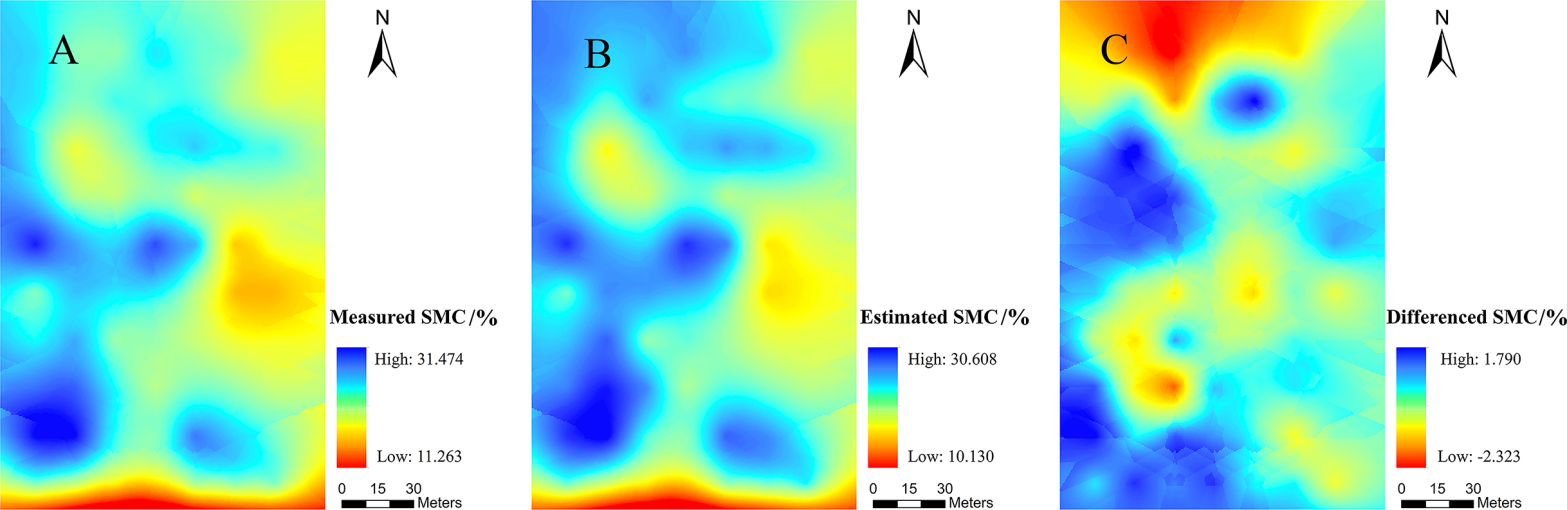
Spatial distribution maps. (A) the measured SMC, (B) the SMC based on PIR prediction, (C) residuals calculated with PIR for prediction of the SMC.

The SMC value in the experimental field was higher in the west than in the east and lower in the south than in the north (Fig. 9). Except for the obvious overestimation in the northern region, the other regions exhibited different degrees of underestimation. The reason for the underestimation in the north might be the fact that the adjacent drainage channel would affect the local SMC. Near the wasteland in the west and south, the lack of vegetation cover might have caused the actual SMC to be low, thereby allowing the possibility of overestimation. Moreover, the maximum residual value was only 2.323%, which indicated that the estimation of SMC via PIR was reasonable at the spatial scale. Therefore, such results confirmed that the PIR model exhibited good performance in spatial simulation.

## Discussion

**Figure 10:**
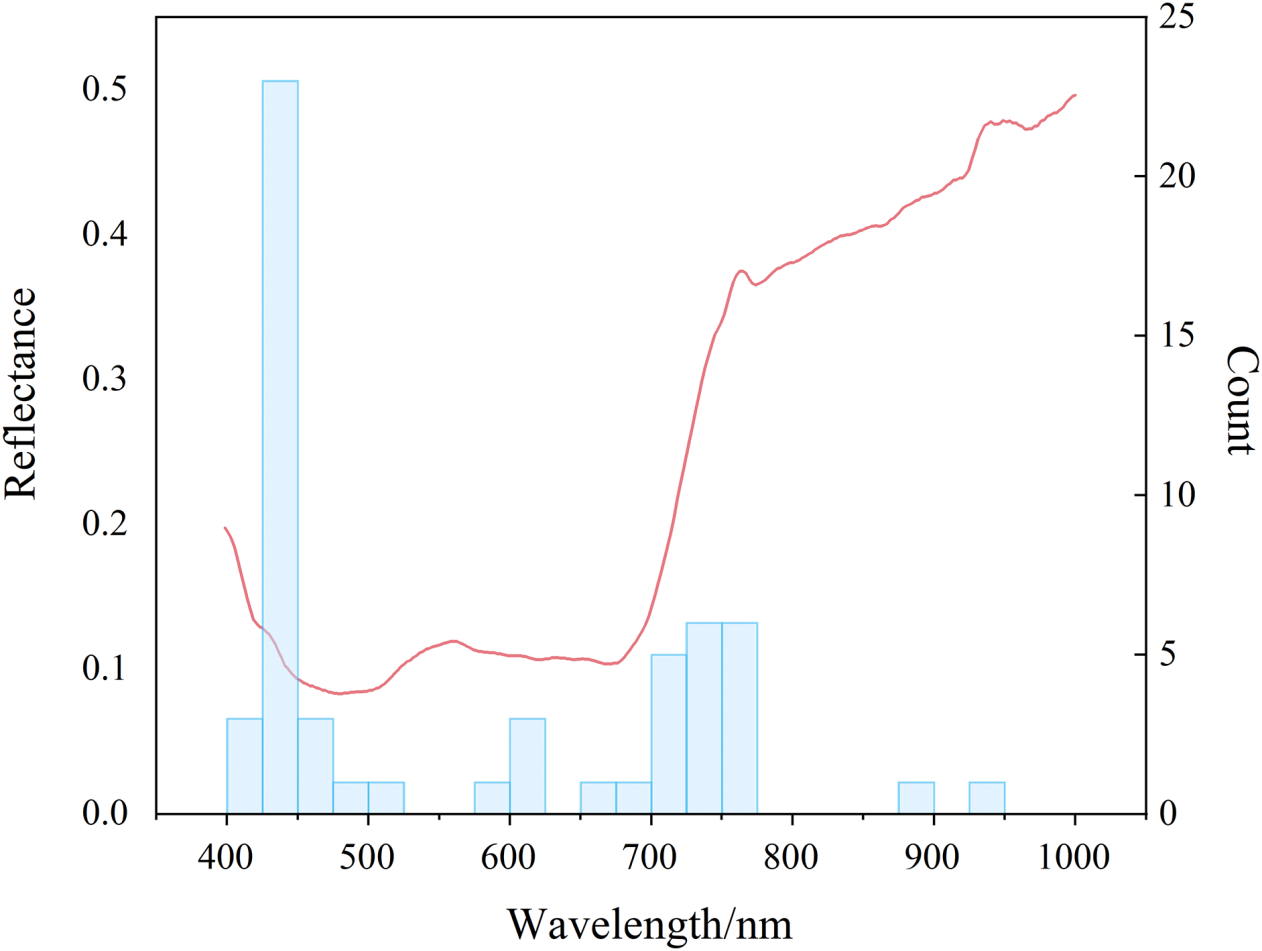
Distribution of sensitive bands. Red lines denote spectral reflectance and blue bars denote distribution frequencies.

The sensitive bands were mainly concentrated in the blue region and the red edge (Fig. 10). There was a certain correlation between SMC and the water content of overlying vegetation leaves. The high and low SMC would affect the water contents of the leaves to different extents and eventually led to changes in the spectral characteristics (Fernández-Novales et al., 2018). Quantitative estimation of SMC based on spectral information on vegetation was feasible when using remote sensing and spectral mechanisms. The bands were concentrated at approximately 420, 440, 460, 700, and 750 nm (Figs. S1–S7). The strong absorption bands of chlorophyll and water in the plants were between 420 and 460 nm (Steidle Neto et al., 2017) and were due to the strong absorption of carotenoids; the strong absorption of chlorophyll in plants near 700 nm, as well as the red edge information of plants and the weak absorption of water, was due to a trough of most vegetation reflectivity (Haboudane et al., 2002). The plant red edge information was near 750 nm, which was the point of strong water and oxygen absorption (Okin et al., 2001). This result suggested the rationality of the index construction. Because the agricultural plants in the arid area had different degrees of water stress, the chlorophyll of the crop canopy fluctuated with the degree of drought, so there was a strong positive correlation between SMC and chlorophyll. Therefore, the developed indices utilized the chlorophyll and moisture response regions (green and red edges) to meet empirical models for estimating SMC from hyperspectral data. The quantitative estimation of SMC based on spectral information of vegetation was feasible when using remote sensing and spectral mechanisms. These results provided a scientific basis for further research on precision agriculture in combination with phenological information. In addition, the results of this study would be conducive to the design of a multiband space-borne remote sensing system for detecting SMC in arid and semiarid regions.

**Figure 11:**
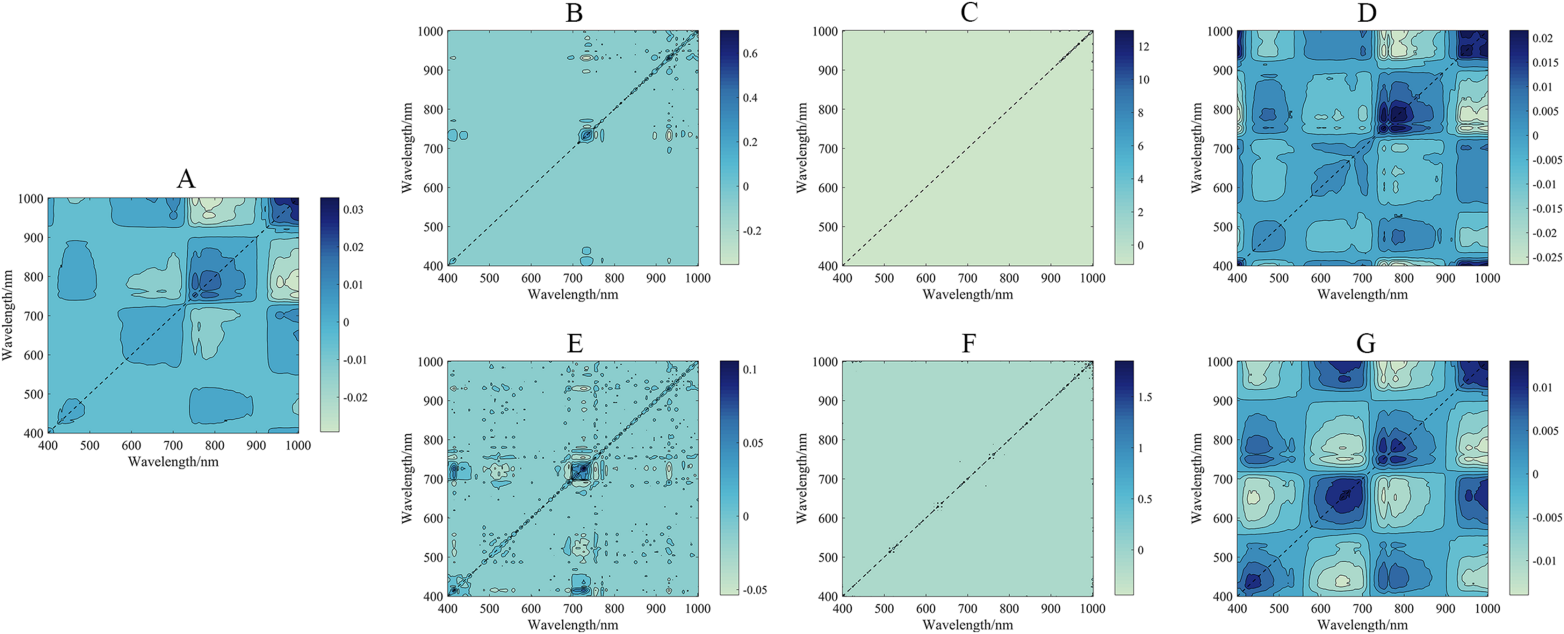
2D synchronized correlation spectrum under different pretreatments. (A) 2D synchronized correlation spectrum based on R. (B) 2D synchronized correlation spectrum based on FDR. (C) 2D synchronized correlation spectrum based on SDR. (D) 2D synchronized correlation spectrum based on CR. (E) 2D synchronized correlation spectrum based on FDA. (F) 2D synchronized correlation spectrum based on SDA. (G) 2D synchronized correlation spectrum based on A.

In this study, six pretreatments (FDR, SDR, CR, A, FDA, and SDA) were used to process the hyperspectral imagery from the UAV and yielded better results than those obtained without pretreatment. However, the one-dimensional spectral information had a deficiency in the expression of spectral information. To discuss and visualize the results of different preprocessing methods, two-dimensional synchronous correlation spectroscopy is introduced in Fig. 11 (Noda, 2016). Two-dimensional synchronous correlation spectroscopy is a correlation intensity map defined by converting one-dimensional spectral data into two independent spectral variables. This process increases the spectral resolution, which allows for the detection of additional spectral information that is difficult to detect in one-dimensional spectra. Obviously, in this study, there were some autocorrelation peaks on the diagonal lines in these two-dimensional synchronous spectrograms. This result suggested the corresponding sensitivity of each functional group to external disturbance and the presence of a synergistic response between the spectra (Hong et al., 2017). The autocorrelation peaks under different preprocessing methods were compared, and this comparison indicated that the FD and SD methods could eliminate a large amount of irrelevant information. These methods result in a narrow range of autocorrelation peaks, but the spectral information of more responses was lost. The performances of R, CR, and A were ranked as A > CR > R. In the two-dimensional synchronous spectrum of A, four autocorrelation peaks appeared, which were located near 450, 670, 740, and 980 nm. This result was similar to the previous discussion on the rationality of the spectral indices. While demonstrating the pretreatment effect, it proved the response mechanisms of the spectral indices.

In general, full-spectrum VIS–NIR data were affected to varying degrees by noises and other factors (Zheng et al., 2016). In arid and semiarid agricultural areas, soil background effects were a major issue for green vegetation property estimates (Ren & Feng, 2014). In theory, soil-adjusted vegetation indices should estimate the aboveground green biomass in our study area better than soil unadjusted vegetation indices; thus, SMC should be estimated with less interference than normal. The four spectral indices based on the R-spectrum data performed best with PI_(446,471)_ (|*r*| = 0.772) (Table 3). Of the four spectral indices based on optimal pretreatment A, the PI_(446,471)_ correlation was still the best (|*r*| = 0.773). These results indicated that the PI used in this study minimized the influence of soil and atmosphere on remote sensing data, dynamically called the reflectivity of each band and better characterized vegetation information.

**Figure 12:**
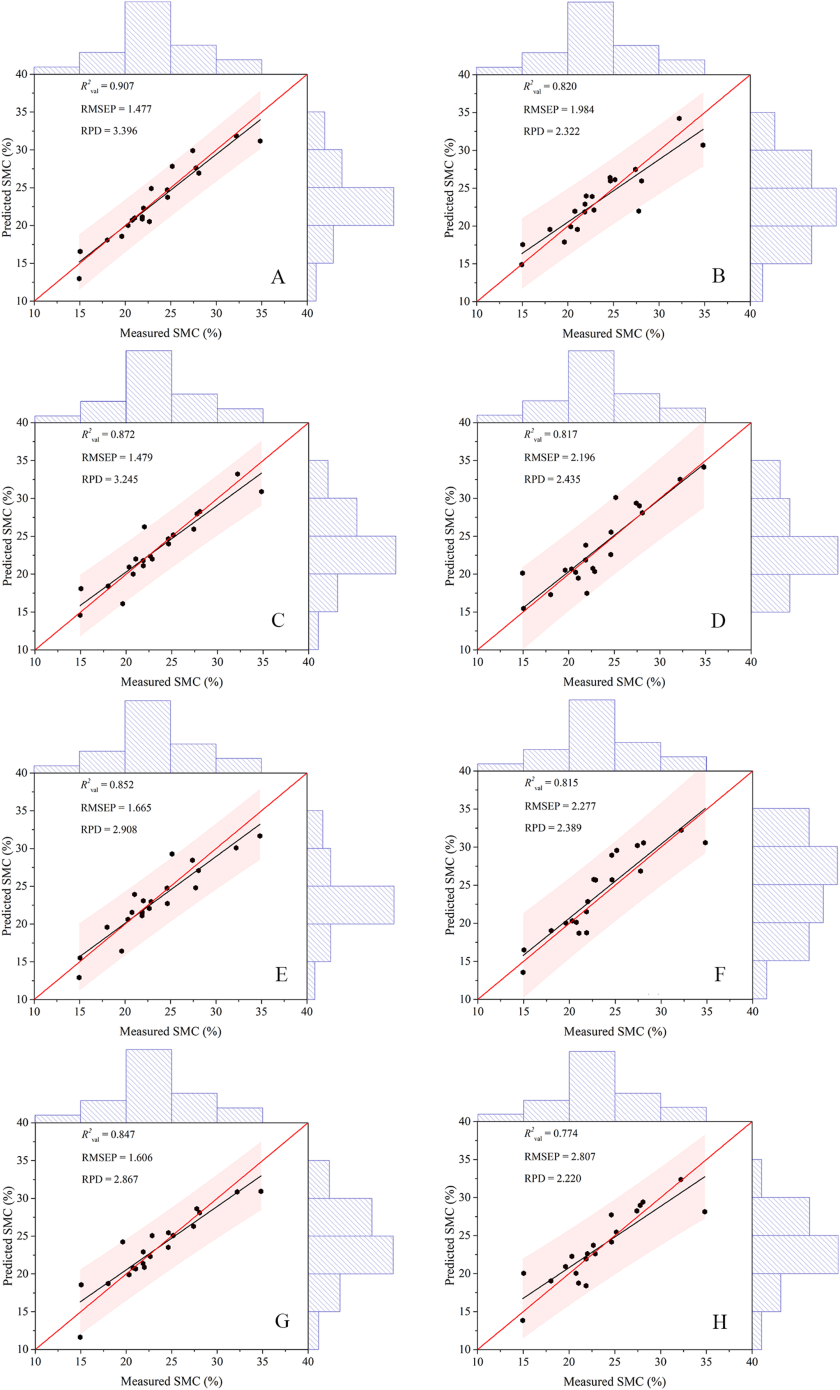
Scatter plots of the measured and predicted SMC based on different modeling methods. (A) The model based on PIR. (B) The model based on RIE. (C) The model based on NDIR. (D) The model based on PIE. (E) The model based on DIR. (F) The model based on NDIE. (G) The model based on RIR. (H) The model based on DIE. The red and black lines in each figure represent the 1:1 and fitted lines, respectively.

Machine learning algorithms have been widely used to estimate soil properties (Ding et al., 2018; Ma et al., 2018; Nawar & Mouazen, 2017). The models in this study yielded different precisions according to different 2D spectral indices. These eight models could achieve excellent modeling results because the spectral indices included in the models utilized the green, red, and red edge information. After pretreatment, the spectral information was effectively extracted, and the model exhibited robust extrapolation ability. However, the calibration set *R*^2^ of the ELM model was higher than that of the validation set, which might result in some defects caused by the randomness of the ELM model (Li et al., 2018); therefore, the fitting effect of the ELM model was not as good as that of the RF model. The validation results of the eight models (Fig. 12) indicated that the scatter points of all models were well distributed along the 1:1 line and the PIR model outperformed the other models. In addition, most models had a scatter line below the 1:1 line. In the arid region, the spatial heterogeneity of soil was significant, which might result in the underestimation of SMC (Thevs et al., 2015; Zhang, Shao & Li, 2017). Studies on the soil properties in arid areas have achieved similar results (Ding et al., 2018; Ma et al., 2018). In recent years, the uncertainties of the ELM method were reviewed by Liu and Lin (Lin et al., 2015; Liu et al., 2015), especially for the different activation functions and subsequent robustness. In general, RF tended to be versatile and flexible, suitable for mining a small subset of features for a small number of samples, and produced unbiased estimates that limited generalization errors (Chen et al., 2019; Lindner et al., 2015). During the training process, the interaction between features could be detected, and the data did not need to be normalized. The RF algorithm has become an effective predictive tool in soil property research because of its high generalization ability. Related studies could provide new ideas for remote sensing monitoring of soil moisture status and a scientific reference for the further development of precision agriculture in arid areas (Belgiu & Drăguţ, 2016).

In this study, the high accuracy of this method provides a new perspective and solution for the integration of remote sensing with the monitoring of soil moisture conditions. Although machine learning algorithms provide improved accuracy, algorithms with many parameters or hyperparameters usually require complex training. The ideal algorithm should have high simulation accuracy and include simple training parameters and low training time requirements (Ding et al., 2018). While unified research on SMC remote sensing estimations based on vegetation spectra has not been established, vegetation spectra would also be affected by factors such as variety, growth period, and soil nutrient status (Casas et al., 2014). However, due to the limitations of weather and means, this study failed to obtain image data from multiple periods, although different growth cycles were considered. Moreover, the migration and generalization abilities of the established SMC machine learning estimation model need to be further improved. Therefore, subsequent research should further explore the intrinsic link between SMC and vegetation hyperspectral reflectance. We thus further developed a large sample of vegetation spectral databases to establish a scientific basis for the quantitative estimation and remote sensing monitoring of precision agricultural parameters such as crop growth, pests, and diseases.

## Conclusion

This research investigated a method that effectively identifies the SMC of agricultural topsoil via UAV hyperspectral imaging in arid regions. Our work proposed a strategy that utilized 2D spectral indices that were more adaptive to special environmental conditions than were traditional spectral indices. Moreover, an effective SMC estimation model was constructed using a machine learning algorithm built on 2D spectral indices. Unmanned aerial vehicle images were processed using different pretreatments to achieve deeper mining of information. Pretreatment absorbance had a strong effect on improving the correlations. The perpendicular index technique exhibited the optimum result (*r* = 0.773) because interference and noise were effectively eliminated. Overall, RF models yielded better predictions than did ELM models. The PIR model possessed the optimal precision for SMC estimation (*R*^2^_val_ = 0.907, RMSEP = 1.477, and RPD = 3.396). The data set that was estimated via PIR maintained the closest statistical characteristics and morphology to the measured data set. The SMC estimated via the PIR model resulted in a digital mapping distribution that was similar to the measured SMC distribution. The optimal model was used to extend the SMC from a single point scale to the area scale to realize remote sensing monitoring of the SMC. The UAV hyperspectral imaging approach described in this study utilizes optimal 2D spectral indices, and the prediction models can supply efficient means to the local environment and agriculture management divisions.

## Supplemental Information

10.7717/peerj.6926/supp-1 Supplemental Information 1 *r*^2^ maps of 2D spectral indices based on R. (A) *r*^2^ maps of R_DI_(479,619)_. (B) *r*^2^ maps of R_RI_(431,446)_. (C) *r*^2^ maps of R_NDI_(431,446)_. (D) *r*^2^ maps of R_PI_(446,471)_. The colorbar illustrates the value of the square of the correlation coefficient (*r*^2^) between SMC and spectral indices, and the *x*-axes and *y*-axes indicate the wavebands of 400–1,000 nm. Dark red portrays a high *r*^2^ between SMC and the spectral indices.

10.7717/peerj.6926/supp-2 Supplemental Information 2 *r*^2^ maps of 2D spectral indices based on FDR. (A) *r*^2^ maps of FDR_DI_(435,746)_. (B) *r*^2^ maps of FDR_RI_(702,724)_. (C) *r*^2^ maps of FDR_NDI_(702,726)_. (D) *r*^2^ maps of FDR_PI_(435,744)_. The colorbar illustrates the value of the square of the correlation coefficient (*r*^2^) between SMC and spectral indices, and the *x*-axes and *y*-axes indicate the wavebands of 400–1,000 nm. Dark red portrays a high *r*^2^ between SMC and the spectral indices.

10.7717/peerj.6926/supp-3 Supplemental Information 3 *r*^2^ maps of 2D spectral indices based on SDR. (A) *r*^2^ maps of SDR_DI_(710,753)_. (B) *r*^2^ maps of SDR_RI_(444,895)_. (C) *r*^2^ maps of SDR_NDI_(417,753)_. (D) *r*^2^ maps of SDR_PI_(653,753)_. The colorbar illustrates the value of the square of the correlation coefficient (*r*^2^) between SMC and spectral indices, and the *x*-axes and *y*-axes indicate the wavebands of 400–1,000 nm. Dark red portrays a high *r*^2^ between SMC and the spectral indices.

10.7717/peerj.6926/supp-4 Supplemental Information 4 *r*^2^ maps of 2D spectral indices based on CR. (A) *r*^2^ maps of CR_DI_(400,446)_. (B) *r*^2^ maps of CR_RI_(431,446)_. (C) *r*^2^ maps of CR_NDI_(431,446)_. (D) *r*^2^ maps of CR_PI_(446,466)_. The colorbar illustrates the value of the square of the correlation coefficient (*r*^2^) between SMC and spectral indices, and the *x*-axes and *y*-axes indicate the wavebands of 400–1,000 nm. Dark red portrays a high *r*^2^ between SMC and the spectral indices.

10.7717/peerj.6926/supp-5 Supplemental Information 5 *r*^2^ maps of 2D spectral indices based on A. (A) *r*^2^ maps of A_DI_(431,446)_. (B) *r*^2^ maps of A_RI_(431,619)_. (C) *r*^2^ maps of A_NDI_(431,619)_. (D) *r*^2^ maps of A_PI_(446,471)_. The colorbar illustrates the value of the square of the correlation coefficient (*r*^2^) between SMC and spectral indices, and the *x*-axes and *y*-axes indicate the wavebands of 400–1,000 nm. Dark red portrays a high *r*^2^ between SMC and the spectral indices.

10.7717/peerj.6926/supp-6 Supplemental Information 6 *r*^2^ maps of 2D spectral indices based on FDA. (A) *r*^2^ maps of FDA_DI_(435,744)_. (B) *r*^2^ maps of FDA_RI_(420,726)_. (C) *r*^2^ maps of FDA_NDI_(513,726)_. (D) *r*^2^ maps of FDA_PI_(435,713)_. The colorbar illustrates the value of the square of the correlation coefficient (*r*^2^) between SMC and spectral indices, and the *x*-axes and *y*-axes indicate the wavebands of 400–1,000 nm. Dark red portrays a high *r*^2^ between SMC and the spectral indices.

10.7717/peerj.6926/supp-7 Supplemental Information 7 *r*^2^ maps of 2D spectral indices based on SDA. (A) *r*^2^ maps of SDA_DI_(579,753)_. (B) *r*^2^ maps of SDA_RI_(440,446)_. (C) *r*^2^ maps of SDA_NDI_(477,753)_. (D) *r*^2^ maps of SDA_PI_(753,946)_. The colorbar illustrates the value of the square of the correlation coefficient (*r*^2^) between SMC and spectral indices, and the *x*-axes and *y*-axes indicate the wavebands of 400–1,000 nm. Dark red portrays a high *r*^2^ between SMC and the spectral indices.

10.7717/peerj.6926/supp-8 Supplemental Information 8 ELM algorithm. ELM algorithm code in matlab

10.7717/peerj.6926/supp-9 Supplemental Information 9 RF algorithm. RF algorithm code in matlab

10.7717/peerj.6926/supp-10 Supplemental Information 10 Spectral information extracted from UAV hyperspectral imagery. Reflectance of samples (*n* = 70)

## Additional Information and Declarations

### Data Availability

The following information was supplied regarding data availability: The RF and ELM algorithms and the raw spectral data are available in the Supplementary Files.
