# Supplementary figures and images for: Combining UAV-based hyperspectral imagery and machine learning algorithms for soil moisture content monitoring
Source: PeerJ. 2019 May 3;7:e6926. doi: 10.7717/peerj.6926 (PMC6501779; doi:10.7717/peerj.6926)

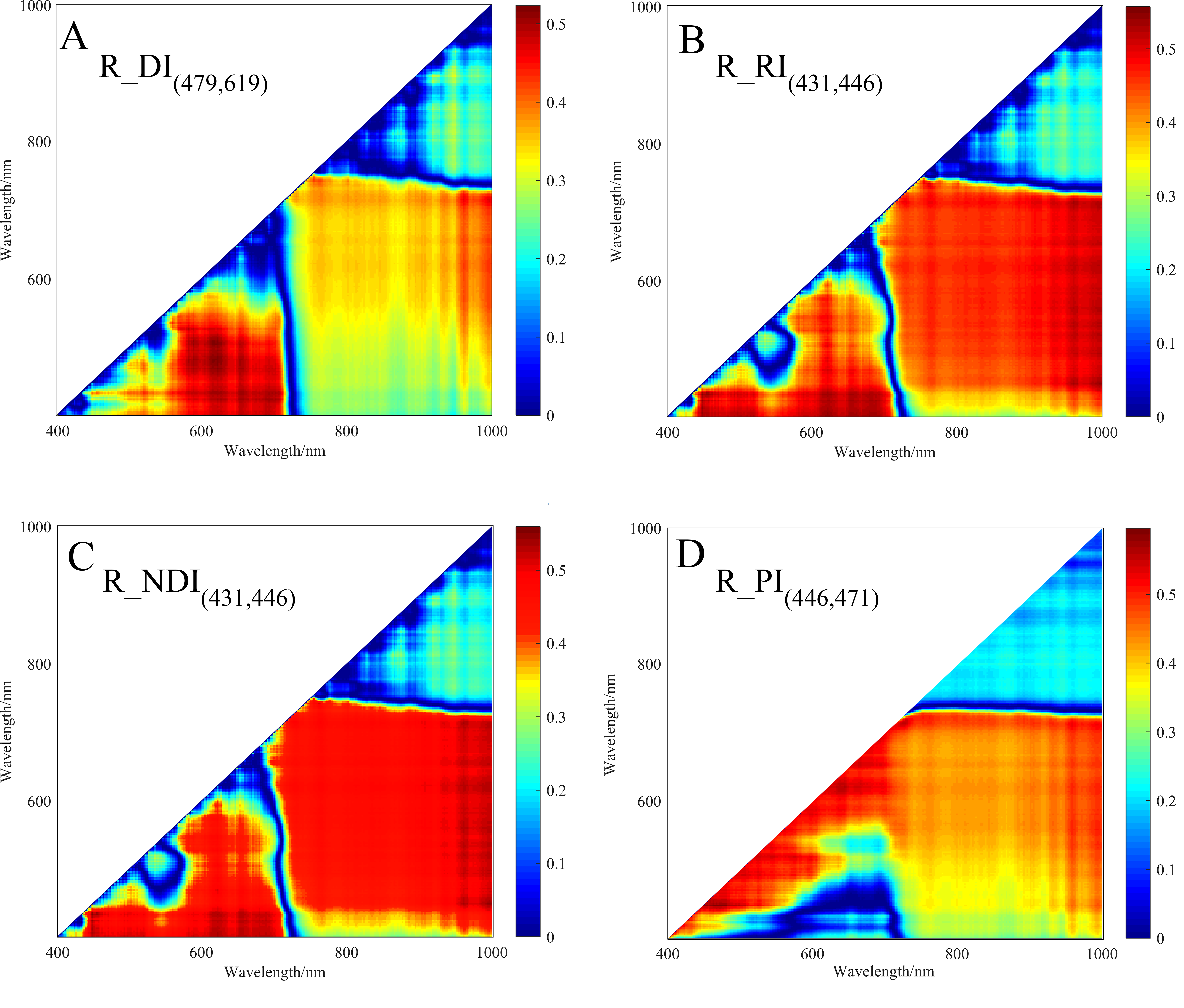

Supplement: Supplemental Information 1 — (A) r2 maps of R_DI(479,619). (B) r2 maps of R_RI(431,446). (C) r2 maps of R_NDI(431,446). (D) r2 maps of R_PI(446,471). The colorbar illustrates the value of the square of the correlation coefficient (r2) between SMC and spectral indices, and the x-axes and y-axes indicate the wavebands of 400–1,000 nm. Dark red portrays a high r2 between SMC and the spectral indices. [file peerj-07-6926-s001.png]

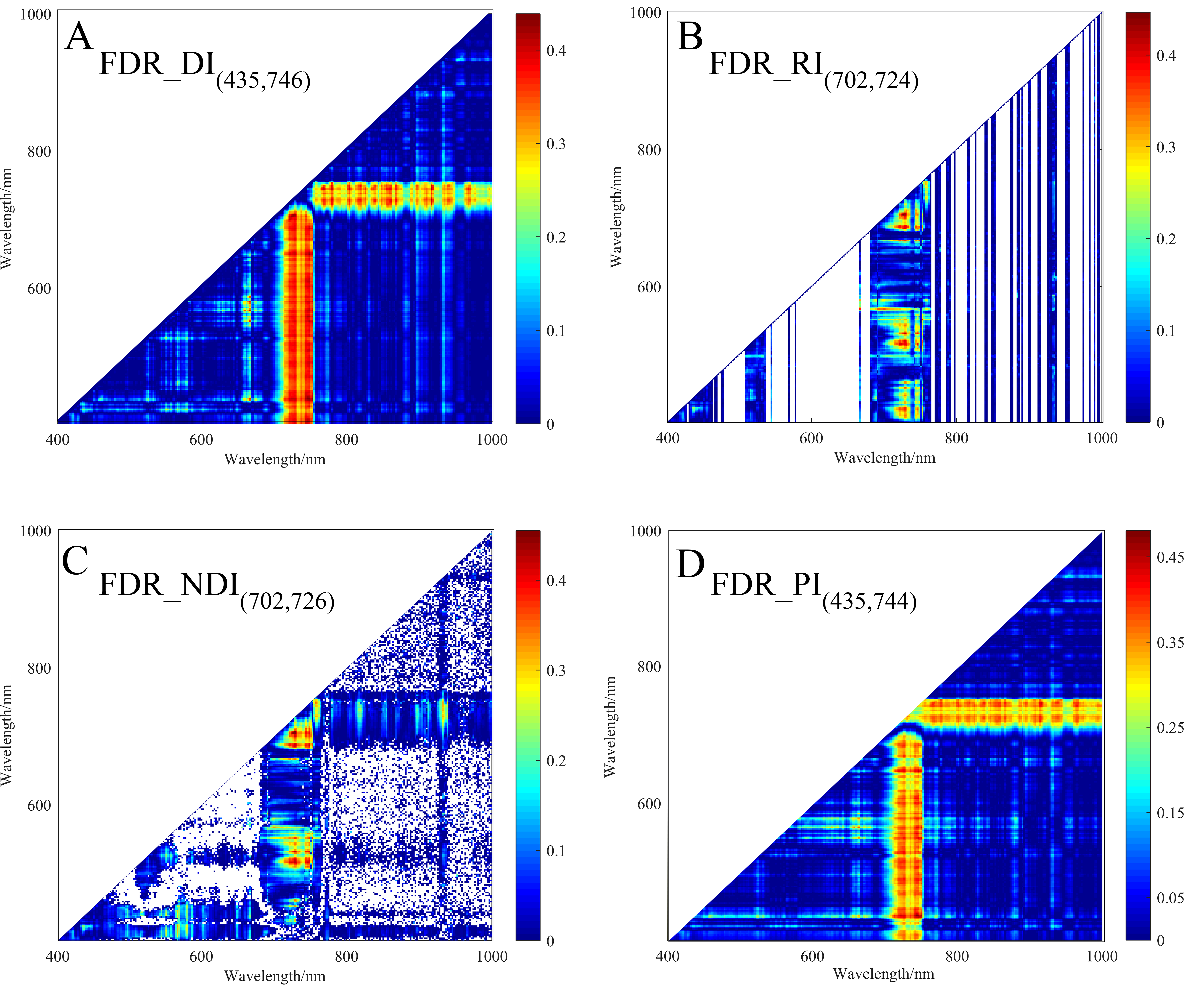

Supplement: Supplemental Information 2 — (A) r2 maps of FDR_DI(435,746). (B) r2 maps of FDR_RI(702,724). (C) r2 maps of FDR_NDI(702,726). (D) r2 maps of FDR_PI(435,744). The colorbar illustrates the value of the square of the correlation coefficient (r2) between SMC and spectral indices, and the x-axes and y-axes indicate the wavebands of 400–1,000 nm. Dark red portrays a high r2 between SMC and the spectral indices. [file peerj-07-6926-s002.png]

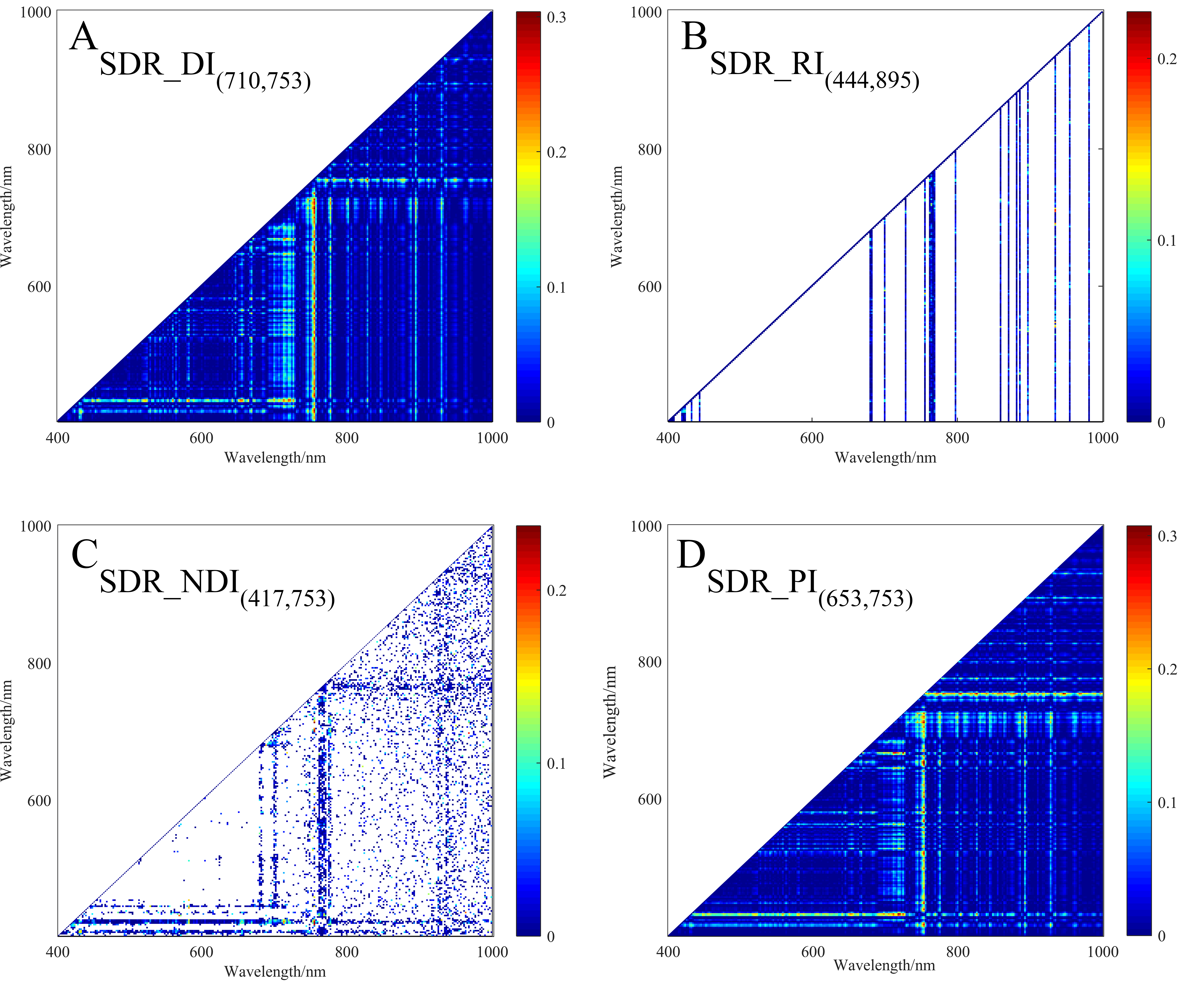

Supplement: Supplemental Information 3 — (A) r2 maps of SDR_DI(710,753). (B) r2 maps of SDR_RI(444,895). (C) r2 maps of SDR_NDI(417,753). (D) r2 maps of SDR_PI(653,753). The colorbar illustrates the value of the square of the correlation coefficient (r2) between SMC and spectral indices, and the x-axes and y-axes indicate the wavebands of 400–1,000 nm. Dark red portrays a high r2 between SMC and the spectral indices. [file peerj-07-6926-s003.png]

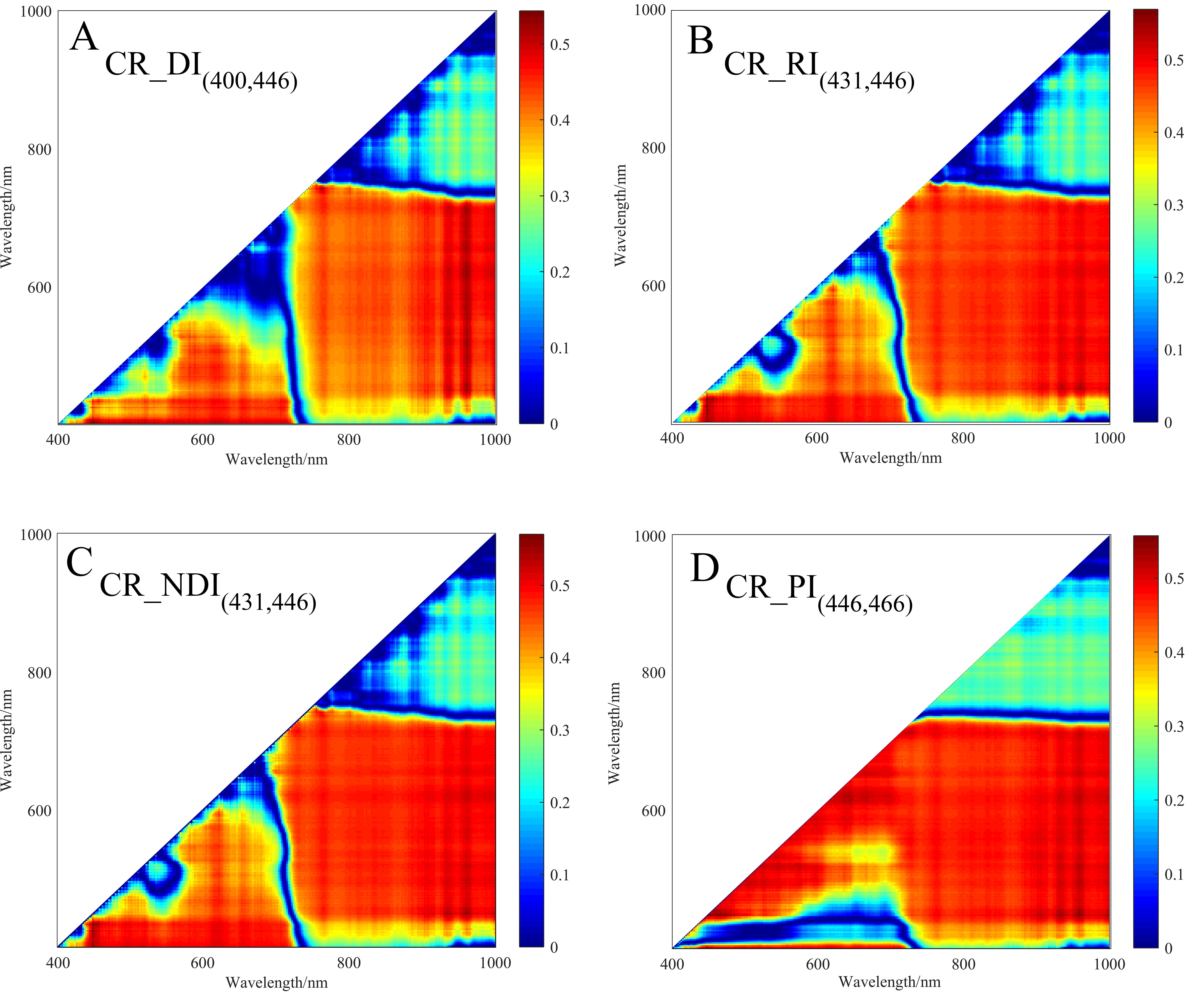

Supplement: Supplemental Information 4 — (A) r2 maps of CR_DI(400,446). (B) r2 maps of CR_RI(431,446). (C) r2 maps of CR_NDI(431,446). (D) r2 maps of CR_PI(446,466). The colorbar illustrates the value of the square of the correlation coefficient (r2) between SMC and spectral indices, and the x-axes and y-axes indicate the wavebands of 400–1,000 nm. Dark red portrays a high r2 between SMC and the spectral indices. [file peerj-07-6926-s004.png]

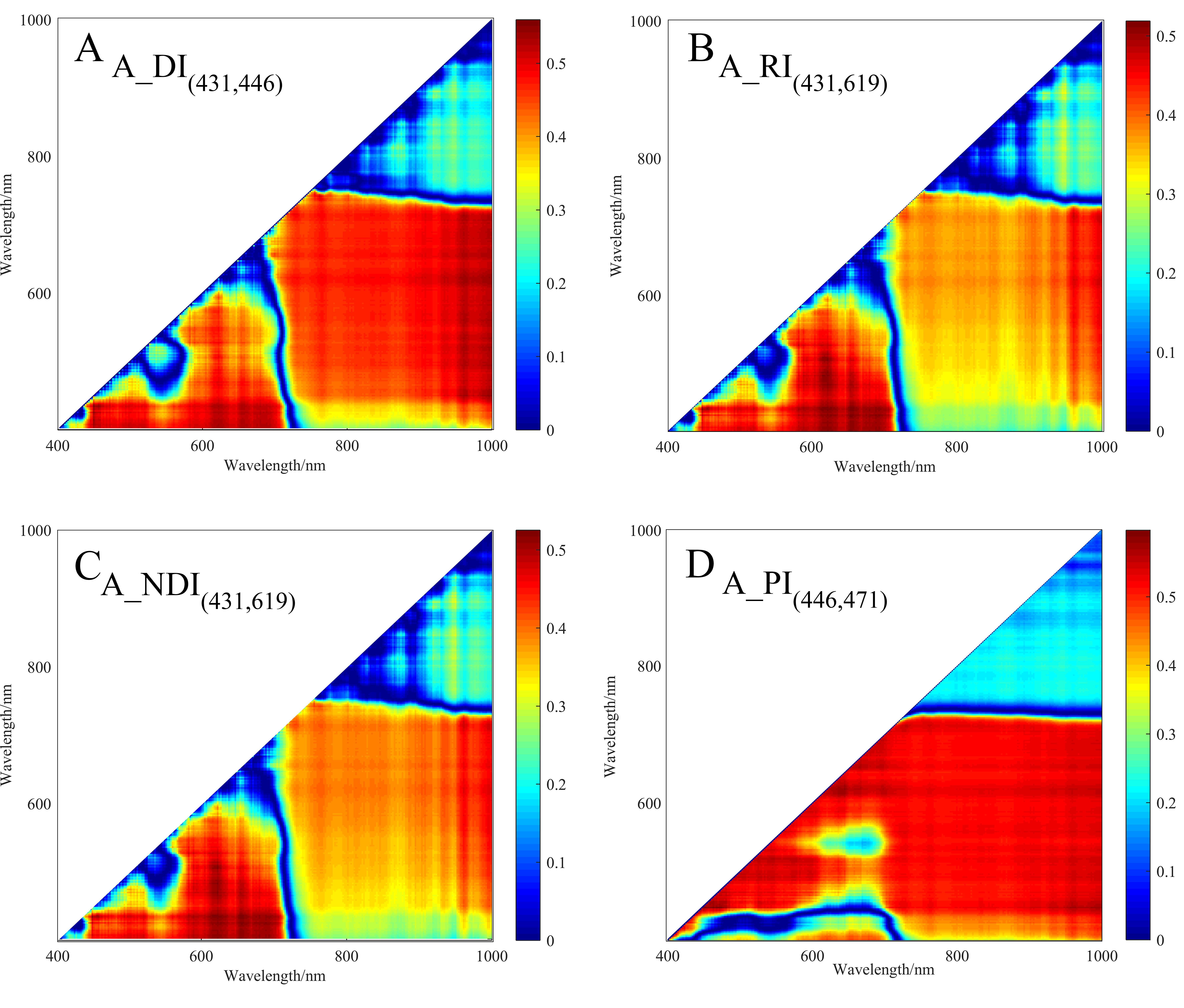

Supplement: Supplemental Information 5 — (A) r2 maps of A_DI(431,446). (B) r2 maps of A_RI(431,619). (C) r2 maps of A_NDI(431,619). (D) r2 maps of A_PI(446,471). The colorbar illustrates the value of the square of the correlation coefficient (r2) between SMC and spectral indices, and the x-axes and y-axes indicate the wavebands of 400–1,000 nm. Dark red portrays a high r2 between SMC and the spectral indices. [file peerj-07-6926-s005.png]

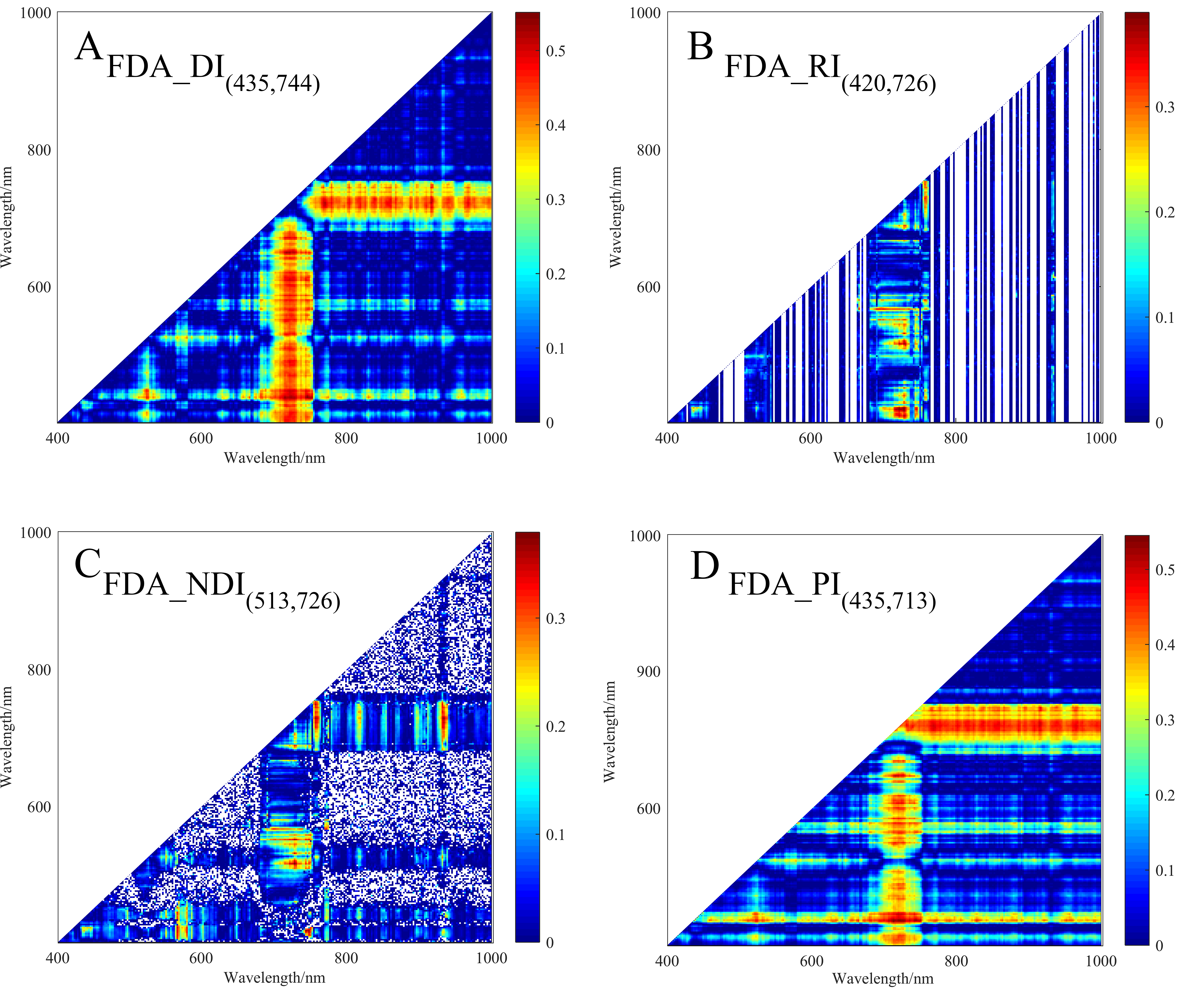

Supplement: Supplemental Information 6 — (A) r2 maps of FDA_DI(435,744). (B) r2 maps of FDA_RI(420,726). (C) r2 maps of FDA_NDI(513,726). (D) r2 maps of FDA_PI(435,713). The colorbar illustrates the value of the square of the correlation coefficient (r2) between SMC and spectral indices, and the x-axes and y-axes indicate the wavebands of 400–1,000 nm. Dark red portrays a high r2 between SMC and the spectral indices. [file peerj-07-6926-s006.png]

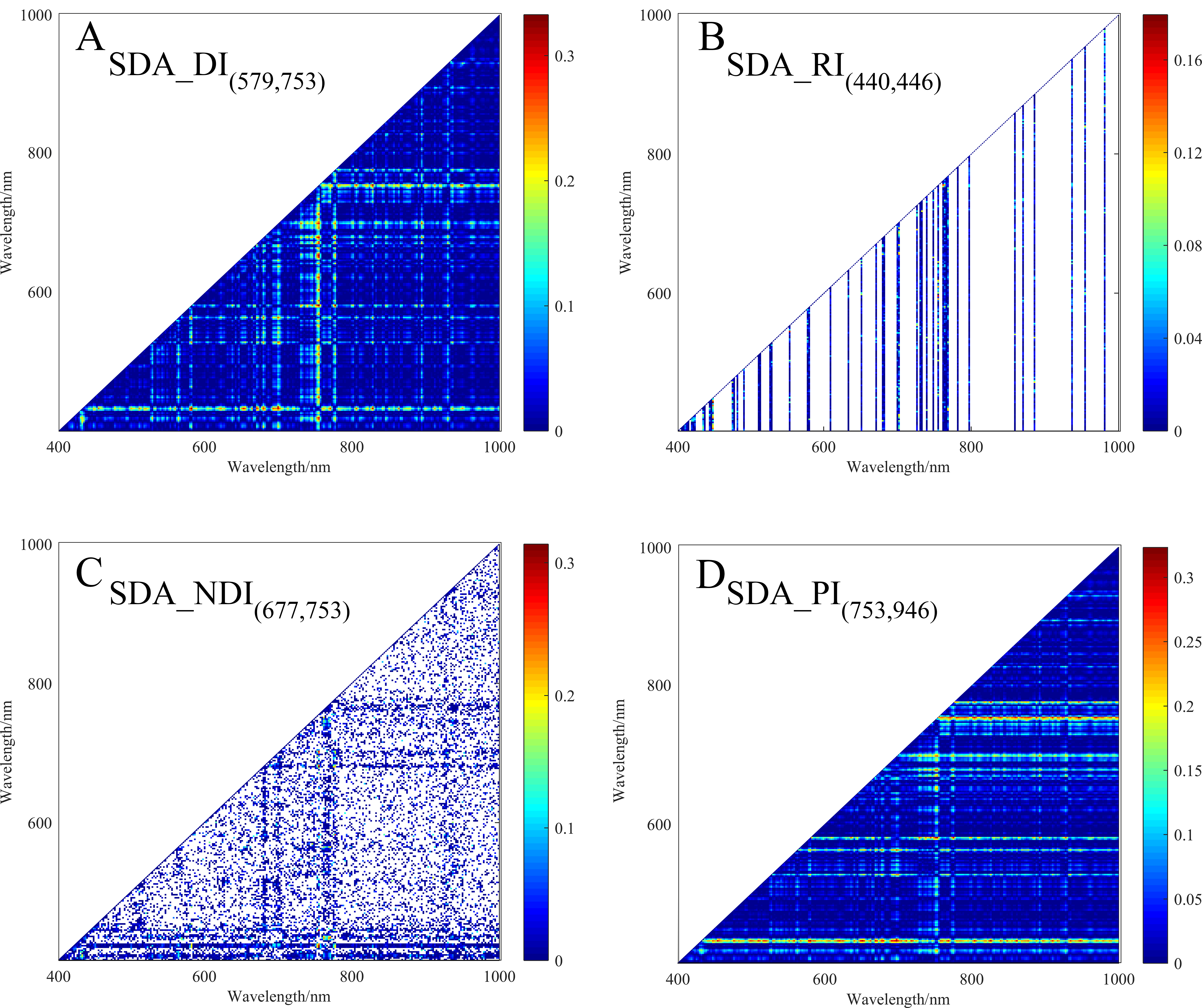

Supplement: Supplemental Information 7 — (A) r2 maps of SDA_DI(579,753). (B) r2 maps of SDA_RI(440,446). (C) r2 maps of SDA_NDI(477,753). (D) r2 maps of SDA_PI(753,946). The colorbar illustrates the value of the square of the correlation coefficient (r2) between SMC and spectral indices, and the x-axes and y-axes indicate the wavebands of 400–1,000 nm. Dark red portrays a high r2 between SMC and the spectral indices. [file peerj-07-6926-s007.png]
